# Anticoagulation Stewardship Program in the DOAC Era

**DOI:** 10.3390/jcm15072597

**Published:** 2026-03-29

**Authors:** Jian Xiong Ng, Su Ching Tan, Pei Lin Koh, Eng Soo Yap

**Affiliations:** 1 Department of Pharmacy, National University Hospital, 5 Lower Kent Ridge Road, Singapore 119074, Singapore; jian_xiong_ng@nuhs.edu.sg (J.X.N.); su_ching_tan@nuhs.edu.sg (S.C.T.); 2 Department of Paediatrics, Khoo Teck Puat National University Children’s Medical Institute, 5 Lower Kent Ridge Road, Singapore 119074, Singapore; pei_lin_koh@nuhs.edu.sg; 3 Department of Paediatrics, Yong Loo Lin School of Medicine, National University of Singapore, 1E Kent Ridge Road, NUHS Tower Block, Level 12, Singapore 119228, Singapore; 4 Department of Laboratory Medicine, National University Hospital, 5 Lower Kent Ridge Road, Singapore 119074, Singapore; 5 Department of Laboratory Medicine, Ng Teng Fong General Hospital, 1 Jurong East Street 21, Singapore 609606, Singapore; 6 Department of Haematology-Oncology, National University Cancer Institute, 5 Lower Kent Ridge Road, Singapore 119074, Singapore

**Keywords:** direct oral anticoagulants, anticoagulation reversal, idarucizumab, andexanet alfa, prothrombin complex concentrate, activated prothrombin complex concentrate, stewardship program, bleeding management

## Abstract

**Background:** Direct oral anticoagulants (DOACs) have transformed antithrombotic therapy but carry significant bleeding risks requiring prompt reversal. Recent regulatory changes have altered the reversal landscape, notably with the withdrawal of andexanet alfa from the U.S. market. Anticoagulation stewardship programs (ASPs) are essential for navigating this evolving environment and optimizing safe use of anticoagulants. **Methods:** This narrative review synthesizes evidence from landmark clinical trials (RE-VERSE AD, ANNEXA-4, ANNEXA-I), contemporary guidelines, emerging literature on reversal agents, and critical regulatory updates including the 2025 U.S Food and Drug Administration (FDA) withdrawal of andexanet alfa. **Results:** Idarucizumab remains the only FDA-approved specific antidote for dabigatran. Following the withdrawal of andexanet alfa, prothrombin complex concentrates (PCCs), both 4-factor and activated are now the primary reversal options for Factor Xa inhibitors, with recent evidence demonstrating comparable hemostatic efficacy. Ciraparantag, a universal reversal agent, is currently in Phase III development. Effective ASPs must now adapt protocols to the post-andexanet era while ensuring timely access to alternative reversal strategies. **Conclusions:** The reversal landscape has undergone a fundamental transformation with the loss of andexanet alfa. Success in DOAC-associated bleeding management now depends on optimizing PCC-based strategies, integrating systematic stewardship approaches, and preparing for emerging universal antidotes. Institutions must urgently update algorithms, ensure PCC availability, and monitor outcomes in this new therapeutic environment.

## 1. Introduction

The emergence of direct oral anticoagulants (DOACs) such as Factor Xa and direct thrombin inhibitors have brought about an evolutionary phase in antithrombotic therapy. DOACs are now commonly favored over warfarin for the treatment and prophylaxis of thromboembolism [1]. However, DOACs also possess bleeding risk: gastrointestinal bleeding occurs more frequently with certain DOACs and still carries mortality risks [2]. Traditional anticoagulants like warfarin, unfractionated heparin, low-molecular-weight heparin are still the mainstay in patients with mechanical heart valves or severe renal impairment [3].

The landscape of DOAC reversal has undergone a dramatic transformation. The development of specific antidotes initially promised to resolve clinical anxiety about managing DOAC-associated bleeding. However, recent regulatory actions have fundamentally altered therapeutic options. This review critically examines the current reversal strategies following these pivotal changes, evaluates the strength of evidence supporting available agents, and provides urgent practical guidance for implementing updated anticoagulation stewardship programs in the post-andexanet era. Importantly, robust anticoagulation stewardship must balance both bleeding and thrombosis risks while integrating oversight of antiplatelet therapy, antifibrinolytic use, and combined antithrombotic regimens to optimize patient outcomes.

## 2. Bleeding Risk Profile of Direct Oral Anticoagulants (DOACs)

The four DOACs labelled for stroke prophylaxis in non-valvular atrial fibrillation (AF) and treatment/prophylaxis of venous thromboembolism (VTE) are dabigatran etexilate (direct thrombin inhibitor), apixaban, rivaroxaban, and edoxaban (Factor Xa inhibitors) [1]. DOACs have shown at least non-inferior efficacy to warfarin in large clinical trials and demonstrated an estimated 50% reduced risk of intracranial hemorrhage compared to Vitamin K antagonists [4,5,6,7]. They have several benefits, including standardized dosing, reduced laboratory monitoring requirements, reduced drug and food interactions, and more predictable pharmacokinetics [1]. Therefore, the risk-benefit balance currently favors DOACs for most indications, and guidelines including the European Society of Cardiology (ESC) 2024 guidelines have given a Class I recommendation to select DOACs over warfarin in eligible AF patients, excluding patients with mechanical heart valves or moderate-to-severe mitral stenosis where warfarin remains the drug of choice [3].

Nevertheless, DOACs still possess significant bleeding risks. The frequency of major bleeding events on DOACs is comparable to that of warfarin, but the difference lies in the distribution of bleeding sites. DOACs have been associated with less intracranial bleeding (ICH), but more gastrointestinal (GI) bleeding, especially in geriatric patients on rivaroxaban or high-dose dabigatran [2]. The net clinical advantage favors DOACs, as the elevated GI bleeding risk is offset by significantly fewer ICH events. However when DOAC-associated bleeding occurs, the consequence can still be dire. A large prospective study of dabigatran-treated patients with major bleeding documented a case fatality of 9% and 17% for GI and intracranial bleeding respectively [8]. Meta-analysis similarly shows high mortality in DOAC-associated ICH [9], highlighting that “less frequent” does not equate benign.

## 3. Perioperative Management of Anticoagulation

The objective of perioperative management of anticoagulation is to reduce intra-procedure bleeding, while minimizing the duration which anticoagulation is withheld to prevent thrombotic complications. Traditionally, this coordination was complicated with warfarin and itself carries bleeding risks. Patients would stop warfarin days before the procedure and commence “bridging” with heparin, before restarting warfarin post-operatively. However, perioperative management has been simplified since the emergence of DOACs, which does not require bridging in most cases, but this requires knowledge of the pharmacokinetics and intra-procedure bleeding risk.

The CHEST perioperative antithrombotic guideline (2022) delivers a holistic framework [10]. Specifically, for DOACs:

- CHEST recommends discontinuing DOAC 1–2 and 3–4 days before low/moderate bleeding risk and high bleeding risk surgeries respectively. Dabigatran should be discontinued earlier (3–4 days) if renal function is impaired, because of its renal elimination. This allows time for the anticoagulant effect to diminish before the planned procedure. No heparin bridging is recommended for DOACs, as bridging does not improve outcomes but increases bleeding risk. The guideline recommends re-initiating DOACs approximately 24 h post-op for low/moderate bleeding-risk procedures, and 48–72 h post-op for high bleeding-risk procedures. The early re-initiation is to limit time off anticoagulation, once sufficient hemostasis is established.

In the context of an emergency surgery (e.g., urgent cardiac surgery, appendectomy due to acute appendicitis), if the surgery timing is within a few half-lives of the last DOAC dose, anticoagulation can dramatically elevate intra-operative bleeding risk. Hence the use of a reversal agent prior to surgery can improve surgical safety. Reversal can be administered in real-time in addition to hemostatic techniques if a patient bleeds excessively during or after surgery.

Perioperative management services, usually operated by pharmacy or anticoagulation services to advise when to discontinue anticoagulants pre-operatively and restart post-operatively, are available in many healthcare institutions. These services can evaluate cases and identify high-risk patients who will benefit from bridging, enhancing patient safety by preventing perioperative bleeding. This ensures consistency and safety. A standardized perioperative anticoagulation management workflow is part of an anticoagulation stewardship initiative. A 2023 report described how adopting standardized peri-procedural protocols and decision support can significantly improve safety [11]. The pre-operative assessment of renal function (to adjust DOAC discontinuation timing if required) and clear instructions on post-operative re-initiation (which should account for surgical hemostasis status) are important considerations when building such protocols.

## 4. Reversal Agents: Current and Emerging

The highlighted reversal agents are idarucizumab, andexanet alfa, ciraparantag, 4-factor prothrombin complex concentrates (PCCs), and activated prothrombin complex concentrates (aPCCs).

Idarucizumab (Praxbind) is a humanized monoclonal antibody fragment developed specifically to bind dabigatran with high affinity, neutralizing its anticoagulant effect. It was the first U.S. Food and Drug Administration (FDA)-approved antidote for a DOAC and is indicated for patients on dabigatran who have life-threatening bleeding or require emergency procedures.

The RE-VERSE AD trial [12] enrolled 503 patients requiring urgent reversal, with 301 experiencing major bleeding and 202 requiring urgent procedures. The study demonstrated that idarucizumab achieved 100% median maximum reversal within 4 h when measured by diluted thrombin time or ecarin clotting time. Clinically, the median time to bleeding cessation was 2.5 h, and 92% of surgical patients had normal or mildly impaired hemostasis. Notably, there were no serious adverse reactions attributed to idarucizumab, and 90-day thrombotic events occurred in 6.3–7.4% of patients, primarily in those not restarted on anticoagulation. The main limitation was rebound anticoagulation observed after 12–24 h in patients with renal impairment due to redistribution of unbound dabigatran.

Idarucizumab is now the sole specific antidote available for DOAC reversal, applicable only to dabigatran. Its uncomplicated dosing (5 g intravenous bolus) and rapid effect make it ideal for emergency use. Institutions must ensure continued availability in emergency departments and critical care pharmacies, particularly as it now represents the only specific reversal option.

Andexanet alfa (Andexxa) is a recombinant modified human Factor Xa protein that works as a high-affinity decoy target for Factor-Xa inhibitors. Through binding, andexanet alfa sequesters these DOACs away from native Factor Xa, restoring the process of the coagulation cascade. Dosing is regimen-based: a “low dose” (400 mg bolus + 4 mg/min infusion) or “high dose” (800 mg bolus + 8 mg/min infusion) depending on the specific DOAC, dose and the time of the last ingested dose. The ANNEXA-4 [13] single-arm study (*n* = 352) and ANNEXA-I [14] randomized trial (*n* = 479) provided evidence for its use. ANNEXA-4 demonstrated a 92% reduction in anti-Xa activity immediately after infusion, with 82% of patients achieving good/excellent hemostasis at 12 h. However, 30-day thrombotic events occurred in 10% of patients, and mortality was 14%. The ANNEXA-I trial [15], which focused specifically on intracranial hemorrhage, found superior hemostatic efficacy versus standard care (PCC) at 12 h, with 94.5% vs. 26.9% reduction in anti-Xa activity. However, there was a concerning thrombotic signal of 10.3% vs. 5.6% with andexanet versus PCC with ischemic stroke occurring in 6.5% vs. 1.5% respectively, although no mortality difference was observed at 30 days.

In 2025, the U.S. FDA announced the withdrawal of andexanet alfa (Andexxa) from the market following a comprehensive safety review [16]. This decision was based on concerns regarding the agent’s risk-benefit profile, particularly the thrombotic signal identified in the ANNEXA-I trial and subsequent post-marketing surveillance data. The FDA determined that the available evidence no longer supported a favorable safety profile for andexanet alfa compared with alternative reversal strategies.

Ciraparantag (Aripazine, PER977) is a synthetic, cationic small molecule that binds multiple anticoagulants (UFH, LMWH, and all DOAC classes) via charge-charge interactions, forming inactive complexes [17]. Phase II studies have demonstrated edoxaban reversal within 10–30 min with effects lasting over 12–24 h without rebound, and no prothrombotic signals have been observed in healthy volunteers [18]. The most common adverse effects are transient flushing and dysgeusia.

The potential advantages of ciraparantag include its versatility as a single agent for all anticoagulants except argatroban [19], which would simplify logistics and reduce inventory burden, particularly for smaller hospitals. Its effects persist longer than andexanet, eliminating rebound concerns. Ciraparantag currently remains investigational, with a Phase III trial (NCT04593784) ongoing for major bleeding and urgent surgery [19]. Regulatory approval will require demonstration of clinical benefit beyond laboratory normalization in real-world bleeding scenarios. Given the withdrawal of andexanet alfa, ciraparantag may assume greater importance if approved, potentially filling the gap for Factor-Xa inhibitor reversal.

Prothrombin Complex Concentrates (PCCs) are lyophilized concentrates of Vitamin K-dependent clotting factors. “4-factor PCC” contains Factors II, VII, IX, X (in combination with Protein C and S). The use of PCCs in DOAC reversal is off-label (use outside of approved product labelling) but are frequently considered when specific antidotes are unavailable [20]. The rationale is to supplement the coagulation cascade with excess clotting factors to overcome the pharmacologic inhibition [21]. 4-factor (4F) PCCs have been demonstrated to normalize DOAC-induced clotting test prolongation partially in laboratory studies, and case series have indicated that PCC can help achieve hemostasis in majority of cases [21]. One retrospective study of PCC use in Factor-Xa inhibitor bleeding demonstrated effective hemostasis in 73% of cases and thromboembolic events in 6.5% [22]. The International Society of Thrombosis and Hemostasis (ISTH) Scientific and Standardization Committee (SSC) in 2024 issued guidance recommending 4-factor PCC as the first-line for life-threatening bleeding with Factor-Xa inhibitors if andexanet alfa is unavailable [20].

Activated Prothrombin Complex Concentrate (aPCC) contains Factors II, IX, X mainly in inactivated forms as well as activated Factor VII and activated Factor VIII coagulation antigen [23] and has been used off-label for DOAC-associated bleeding. A recent retrospective cohort study comparing aPCC versus 4F-PCC for apixaban- and rivaroxaban-associated major bleeding provides important new guidance in the post-andexanet era [24]. Among 293 patients (252 receiving aPCC, 41 receiving 4F-PCC), baseline characteristics were similar except for more intracranial hemorrhage in the 4F-PCC group (75.6% vs. 46.8%). Hemostasis was achieved in 77.0% of aPCC-treated and 70.7% of 4F-PCC-treated patients, with no statistically significant difference. Thromboembolic events were infrequent in both groups (1.6% vs. 4.9%), with no significant difference. These results demonstrate that aPCC and 4F-PCC achieve comparable hemostatic outcomes with low thromboembolic event rates in Factor-Xa inhibitor-associated major bleeding.

The comparable hemostatic efficacy between aPCC and 4F-PCC likely reflects different mechanistic contributions. While aPCC contains activated Factor VII (FVIIa) that can directly activate Factor X downstream of the inhibited Factor Xa, 4F-PCC contains Protein C and Protein S as important regulatory components that provide natural anticoagulant balance. Protein C, when activated by the thrombin-thrombomodulin complex, inactivates Factors Va and VIIIa, while Protein S serves as its cofactor. The observed lower thromboembolic event rate with aPCC (1.6% vs. 4.9%) in this study requires cautious interpretation given its retrospective design and baseline imbalances (notably more intracranial hemorrhage in the 4F-PCC group). Potential explanations include differential dosing (aPCC may achieve hemostasis at lower effective procoagulant doses due to pre-activated factors), physician selection bias, or pharmacokinetic differences in factor half-lives. However, this single study does not establish aPCC superiority regarding thrombotic safety, and both agents remain reasonable options in the post-andexanet era.

Overall, both aPCC and 4F-PCC have comparable reported hemostatic efficacy, but institutional protocols may preferentially adopt one strategy based on formulary availability and cost considerations. In our institution, 4F-PCC is the preferred agent for this reason.

Adjunctive therapies such as charcoal and tranexamic acid can be considered as part of the reversal strategy for DOACs (Table 1 and Supplementary File S3).

## 5. Implications for Anticoagulant Selection in High-Risk Patients

The current reversal landscape where idarucizumab provides specific reversal for dabigatran while Factor-Xa inhibitors rely on off-label PCC-based strategies creates important considerations for anticoagulant selection in high-risk patients. Several factors may influence this decision.

Idarucizumab offers rapid, complete, and predictable reversal without prothrombotic signals, potentially favoring dabigatran in patients at high bleeding risk (e.g., prior intracranial hemorrhage, frequent falls, planned invasive procedures).

Factor-Xa inhibitors, despite reversal limitations, have favorable bleeding profile. Apixaban demonstrates lower gastrointestinal bleeding rates compared to dabigatran and once-daily regimens (rivaroxaban, edoxaban) may improve adherence in selected populations. In addition, Factor-Xa inhibitors have fewer P-glycoprotein interactions than dabigatran. Therefore, reversal availability should be one factor amongst many in anticoagulant selection, particularly for patients with previous major bleeding or those likely to require urgent surgery. However, we caution against systematic preferential prescribing of dabigatran solely based on reversal availability, as net clinical benefit depends on multiple patient-specific factors. ASPs should ensure clinicians understand these trade-offs without inappropriately favoring one agent class.

## 6. Laboratory and Workflow Considerations in Reversal

**Figure 1:**
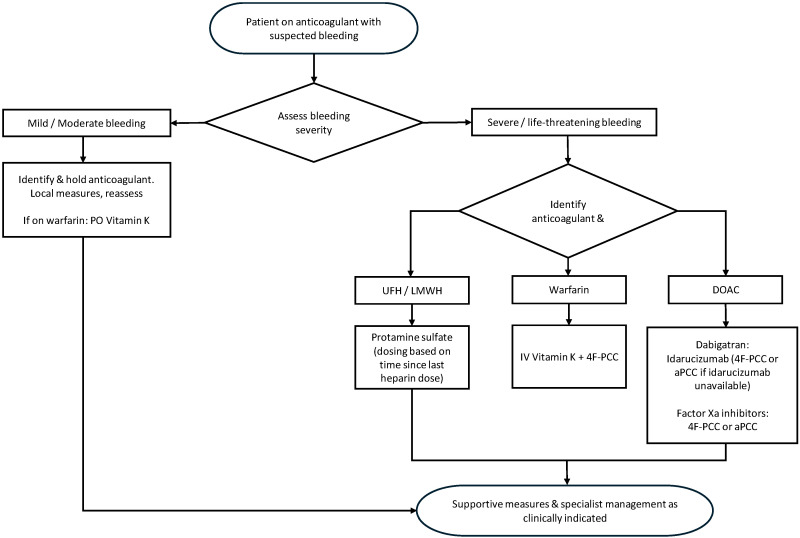
Anticoagulation reversal decision map.

Laboratory readiness and robust clinical workflows (as illustrated by an anticoagulation reversal decision map in Figure 1) are key to harmonizing anticoagulation reversal into clinical practice so that decisions are made and executed without delay. In this section, we explore the role of laboratory assays in guiding reversal (summarized in Table 1), usual turnaround time, point-of-care testing developments, and how institutions refine protocols to facilitate reversal in emergency situations.

A standard laboratory panel encompasses prothrombin time, international normalized ratio (PT/INR) and activated partial thromboplastin time (aPTT). DOACs affect these results in a variable manner:

- Dabigatran prolongs aPTT and thrombin time significantly if levels are high. A normal thrombin time likely indicates an insignificant level of dabigatran.
- Factor-Xa inhibitors cause a dose-dependent prolongation of PT, especially rivaroxaban, but a normal PT does not exclude their presence. While an anti-Xa assay calibrated for specific DOACs is the best indicator, the long turnaround time renders it impractical to wait for drug levels before reversal in an emergency.

Anti-Xa levels calibrated to LMWH or specific DOACs can be measured using chromogenic assays. Dabigatran levels can be measured by a dilute thrombin time or ecarin clotting assay. These tests can guide reversal by determining if a substantial level of anticoagulant is detected:

- If a DOAC level is low (<30 ng/mL), emergency procedures may proceed without reversal; a mild bleed may not require the administration of an antidote.
- If levels are high, it supports the approach for an aggressive reversal.

However, DOAC level testing may not be routinely available in many institutions. Some send out to external laboratories, which has little utility in emergency situations. Therefore, emphasis has been placed on point-of-care testing.

The DOAC Dipstick is a qualitative urine assay that rapidly detects the presence of DOACs, including factor-Xa inhibitors and direct thrombin inhibitors, and has been shown to reliably exclude clinically relevant plasma concentrations (≥30 ng/mL) in acute settings [25,26]. A multicenter prospective registry study is currently underway to evaluate its accuracy and clinical utility in guiding thrombolysis decisions in acute stroke, specifically assessing whether a negative urine test can safely permit administration of tissue plasminogen activator (tPA) [27]. Feasibility studies in acute stroke patients demonstrate that a negative urine DOAC test correlates with low plasma DOAC levels and would likely expand eligibility for thrombolysis.

For bleeding patients, a positive urine DOAC test may indicate the need for reversal agents, while a negative test especially if sufficient time has elapsed since the last dose may help avoid unnecessary reversal, as supported by expert consensus algorithms [20,28]. ISTH recommends reversal for DOAC levels >50 ng/mL in serious bleeding, and >30 ng/mL for high-risk procedures [20]. Point-of-care devices measuring whole blood clotting time, such as Hemochron, have also been validated to detect DOAC effect and guide emergency management, though their adoption remains limited [29,30]. These rapid bedside tools are not yet standard in clinical practice but are under active investigation and may become integral to emergency care workflows in the future.

## 7. Anticoagulation Stewardship Services

The concept of anticoagulation stewardship emerged as a critical response to the significant and often preventable harm associated with anticoagulant use, which is consistently ranked among the top causes of medication-related injuries [31,32]. Anticoagulation Stewardship Programs (ASPs) aim to deliver safe, effective and judicious use of anticoagulants across healthcare settings [33,34,35]. We explore the evolution of these programs, current models of implementation, and discuss how they can tackle future challenges, including integrating newer agents.

Anticoagulation stewardship is defined by the Anticoagulation Forum as a “coordinated, efficient, and sustainable system-level initiatives designed to achieve optimal anticoagulant-related health outcomes and minimize avoidable adverse drug events through the application of optimal evidence-based care, appropriate prescribing, dispensing and administration of anticoagulants and related agents, and provision of appropriate patient monitoring and clinical responsiveness” [36]. Anticoagulation stewardship originally emerged in the form of anticoagulation clinics, typically pharmacist-run outpatient services to adjust warfarin doses based on INR readings [37]. The introduction and rapid adoption of DOACs marked a significant inflection point for anticoagulation stewardship. As DOACs do not require similarly intensive lab monitoring, such oversight on DOACs therapy was not given as much importance [36]. However, the importance of global oversight became apparent as DOACs were implicated in 40% of emergency department visits for anticoagulant-related bleeding [38]. Proper DOAC dosing (including adjustments for renal function), monitoring adherence, perioperative management, and tackling drug interactions require expert coordination. With increasing number of anticoagulants and reversal agents in the market, a comprehensive strategy to prevent errors and improve outcomes is critical.

The Joint Commission (TJC) published the R3 Report Issue 19 in December 2018, which announced that effective from 1 July 2019, new requirements would be applicable to all accredited nursing care centers and medical centers. These requirements are codified as National Patient Safety Goal NPSG.03.05.01 and mandate protocols for anticoagulant therapy, including the use of evidence-based guidelines for anticoagulation reversal and perioperative management [39,40].

## 8. Current Implementation Models

An ideal ASP team requires contribution from multiple stakeholders, including pharmacists, hematology and cardiology physicians, nurses, quality officers and lab or information technology (IT) experts. A 2023 study on hospital-based multidisciplinary anticoagulation stewardship placed importance on patient-centered care and informed decision making, enabled by a formal team-based strategy [33].

Scope of Activities [36]:

- Policy and guideline development: Devising institutional protocols for anticoagulation therapy including dosing, monitoring and reversal [41].
- Education: Equipping clinicians and nurses with knowledge in anticoagulant management and administration is essential for patient safety. Studies have shown that education initiatives to healthcare professionals reduce anticoagulant-related medication errors [42,43].
- Real-time clinical consultation: Some ASPs provide a consult service, for example, to clinicians who require guidance on anticoagulation dosing in unique cases [33,44]
- Transition of care coordination: Ensuring that patients are given appropriate follow-ups when discharged with anticoagulants, especially warfarin. This reduces the risk of readmissions due to thrombotic events or bleeding complications resulting from non-adherence or incorrect medication administration after discharge [33].
- Perioperative case management: Many ASPs provide perioperative anticoagulation management service, where they receive referrals for patients on anticoagulants listed for surgeries and provide an appropriate perioperative management plan for the anticoagulation therapy. This ensures consistency and reduces unnecessary variability or last-minute cancellations due to lack of coordination regarding anticoagulation management plans pre-op [33].
- Monitoring outcomes and quality improvement: TJC mandates that ASPs track data on adverse events such as the number of heparin-induced thrombocytopenia cases, incidence of supratherapeutic INRs resulting in bleeds, or serious DOAC-associated bleeding events and their management. These cases are evaluated for adherence to protocols and identify key areas for improvements [40].
- Technology and alerts: The IT team can be involved to roll out electronic health records (EHR) alerts, such as an alert that triggers correction when a DOAC is prescribed at a wrong dose for a specific renal function. They can also assist in the integration of clinical decision support, such as default order sets that include appropriate reversal orders if required [33].

Pharmacist-led check of medication appropriateness (CMA) intervention, which involved daily review of anticoagulant prescriptions using clinical decision rules, resulted in a 70% immediate relative reduction in residual potentially inappropriate prescriptions (PIPs) for anticoagulants. The intervention enabled pharmacists to identify and address inappropriate dosing and indications before errors reached patients, with 75% of recommendations accepted by prescribers. This demonstrates that daily pharmacist review of anticoagulant orders creates actionable opportunities to prevent medication errors and adverse events through timely intervention, supporting safer anticoagulant prescribing in hospitalized patients [45,46]. These findings are consistent with broader evidence that pharmacist-led stewardship and structured medication review are effective strategies for reducing clinically significant drug-related problems and improving medication safety outcomes in patients receiving antithrombotic therapy [45,46]. In addition, ASP targets can be aligned with wider institutional goals. For example, major bleeding rates on anticoagulants can be monitored as a safety metric, with the aim of reducing preventable bleeds such as overdosing an elderly patient on a DOAC.

## 9. Future Directions

As the anticoagulation therapy landscape continues to evolve with new anticoagulants and reversal agents emerging, ASPs will:

- Develop unified reversal algorithm: A single algorithm that outlines the strategy to any anticoagulant-related bleeding will be developed. Having a single, unified algorithm will help to reduce confusion and delays during bleeding emergencies. A unified anticoagulation reversal toolkit may be implemented, such as a checklist in an EHR order set.
- Monitoring and feedback: Data analytics will be increasingly utilized to identify patterns. For example, tracking reversal agent usage patterns and clinical outcomes and providing targeted feedback to clinicians who deviate from best practices or institution protocols.
- Performance metrics alignment: Hospitals may establish measurable targets, such as “For any anticoagulated patient with ICH, reversal agent administration within 60 min in >90% of cases” or “100% of warfarin patients with critical INR > 10 get Vitamin K within X time”. These can be implemented as quality improvement metrics, advanced by stewardship.
- Extension to antithrombotic stewardship: Many patients are on multiple antithrombotic drugs (including both anticoagulants and antiplatelets). The deprescribing of aspirin in a patient on warfarin for atrial fibrillation with no additional indication for aspirin, can reduce bleeding without an increase in thrombotic events. This comprehensive approach examines all blood-thinning therapies a patient is on, optimizing the combination.
- Patient education and engagement: A greater emphasis on patient involvement will be part of the future of ASP. This includes education on the importance of compliance to therapy, identification of bleeding and thrombotic symptoms early, and when to seek immediate medical attention. TJC mandates patient education on anticoagulation. ASPs develop education materials and teach-back methods to ensure good understanding of therapy in patients.

Future development of anticoagulation stewardship programs will likely focus on improved integration of clinical, laboratory, and pharmacologic data to support individualized decision-making. Advances in EHR and clinical decision-support systems may enable real-time risk stratification for bleeding and thrombosis. In addition, emerging artificial intelligence–based approaches may facilitate the development of patient-specific algorithms that integrate clinical variables, laboratory markers, and pharmacokinetic data to optimize anticoagulation management. Such approaches could enhance precision in anticoagulation stewardship and help improve patient outcomes.

## 10. Practical Recommendations

We propose the following practical recommendations for healthcare institutions aiming to harmonize antithrombotic therapy with anticoagulation reversal:

1. Establish a multidisciplinary ASP: Hospitals should convene an anticoagulation safety committee (including cardiology, neurology, hematology, pharmacy, surgery/anesthesia, and quality officers) to devise and oversee the adherence to protocols. This program should be empowered to implement guidelines (e.g., CHEST, ESC) into institutional practice and ensure compliance with TJC NPSG requirements. This will involve regular multidisciplinary meetings to evaluate anticoagulant-related adverse events and updating of protocols.
2. Develop unified reversal algorithm: A single, easy-to-follow algorithm covering all anticoagulants (warfarin, heparins, DOACs) for managing anticoagulant-associated bleeds should be developed. This should be stratified by severity and include dosing recommendations. The protocol should then be distributed widely, such as posting on intranet, emergency departments, and even as an EHR order set. A “code hemorrhage” checklist based on the algorithm can guide acute responses. An example of our institution’s guide is in Supplementary File S1.
3. Stock and strategically deploy reversal agents: Approved specific antidotes such as idarucizumab should be made available in areas likely to require them (emergency departments, intensive care units, pharmacy after-hours kit) to minimize drug turn-around time. If cost or resource constraints limit usage, the criteria for use should be clearly defined to avoid hesitation or confusion.
4. Utilize laboratory support effectively: Laboratories should validate and offer assays that assist in the evaluation of anticoagulation, including DOAC-calibrated anti-Xa levels and thrombin time or ecarin clotting time for dabigatran. The capability to measure drug levels within 60 min can guide cases (e.g., late-presenting bleeds or uncertain adherence) even if treatment should not be withheld for these results in emergencies. Point-of-care tests should be utilized if available, such as using a DOAC urine dipstick in the emergency department to swiftly assess the anticoagulation status of stroke patients. Baseline coagulation labs (e.g., International Normalized Ratio INR, activated partial thromboplastin time aPTT) should be taken for all bleeding patients and resulted immediately to guide management by identifying an unsuspected coagulopathy or combined antiplatelet use.
5. Protocolize perioperative anticoagulation management: These institutional protocols that guide clinicians on the perioperative anticoagulation management should be built on current evidence-based guidelines, and pre-operative holding intervals should be standardized for each DOAC customized to renal function and surgical bleed risk and be included in pre-operative checklists or order sets. Post-operative reinitiation protocols should be given equal importance to mitigate thrombotic risk after sufficient hemostasis has been achieved. Heparin bridging should be reserved for truly high-risk patients (e.g., mechanical valves, recent venous thromboembolism) and should involve the stewardship service or thrombosis specialist to coordinate timing and provide reversal if required. This limits variability and prevents both bleeding and thrombotic events. Our institution’s peri-operative anticoagulation protocol is in Supplementary File S2.
6. Monitor outcomes and adverse events: A system to track and evaluate anticoagulation-related outcomes such as the frequency of major bleeds, reversal agent use, thrombotic events after reversal, and perioperative thromboembolism should be implemented. These reviews can help to improve current processes and improve quality of care. Appropriate prescribing, such as correct DOAC dosing based on patient factors, should be evaluated. These findings should be delivered to hospital quality committees and if applicable, on public quality dashboards.
7. Align with performance and safety metrics: Established metrics should be adopted to measure the impact of ASP. Patient-centered outcomes such as reduced in-hospital mortality for anticoagulated trauma patients after implementing rapid reversal protocols should be monitored.
8. Educate clinicians and patients continuously: Frontline clinicians (emergency physicians, surgeons, neurologists, etc.) can be reminded of the reversal protocols and guideline updates. Mock drills for critical scenarios such as a DOAC patient with ICH to reinforce steps. Quick reference guides can be disseminated or integrated into information buttons in the EHR for guidance. It is also important to ensure that patients possess good understanding of their anticoagulant therapy and the importance of communicating it in emergencies. Patients can be empowered with wallet cards or medical alert identification cards indicating their anticoagulant, which can save precious time in an emergency if they are unable to provide history.

## 11. Conclusions

The integration of antithrombotic therapy with anticoagulation reversal is a sign of critical advancement in optimizing patient safety in the current ever-developing anticoagulation landscape. We now possess effective anticoagulants that improve patient outcomes in AF, VTE and other conditions, in combination with effective reversal agents when bleeding happens. With judicious application of guidelines, use of reversal agents as appropriate, and systematic stewardship efforts, clinicians can maximize the benefits of anticoagulation while limiting its risks. The evolution from warfarin’s crude reversal with plasma to today’s specific antidotes exemplifies progress in pharmacology, but it is the systems and protocols around their use that determine patient outcomes. By implementing effective anticoagulation stewardship programs, hospitals can ensure that when bleeding emergencies occur, the clinician is prepared with a plan, the appropriate antidote is administered without delay, and the patient is steered towards the best possible outcome. The future of antithrombotic care will likely feature safer anticoagulants, more reversal options, and smarter decision support systems, but the core principles of rapid reversal of life-threatening bleeding, careful peri-operative management, and multidisciplinary oversight will remain the mainstay of high-quality care.

## Figures and Tables

**Table 1:** Guidance on the laboratory detection and reversal strategies for the various anticoagulants.

Anticoagulant	Laboratory Detection	Specific Antidote	Non-Specific Reversal	Adjunctive Therapy
Warfarin	PT/INR (therapeutic: 2.0–3.0)	Vitamin K (IV 5–10 mg)	4F-PCC (25–50 U/kg)	FFP if PCC unavailable
Unfractionated heparin	aPTT, anti-Xa activity	Protamine sulfate (1 mg per 100 U heparin)	N/A	N/A
Low molecular weight heparin	Anti-Xa activity (LMWH-calibrated)	Protamine (partial reversal, 1 mg per 1 mg enoxaparin)	N/A	N/A
Dabigatran	aPTT, TT, ECT, dilute TT	Idarucizumab 5 g IV	4F-PCC (50 U/kg) or aPCC (50–80 U/kg)	Hemodialysis (removes ~60%) Consider: (a) Charcoal (if within 2 h of the last dose of DOAC) (b) Tranexamic acid
Apixaban	PT (variable), anti-Xa (specific)	None (andexanet withdrawn)	4F-PCC (25–50 U/kg) or aPCC (50–80 U/kg)	Consider: (a) Charcoal (if within 2 h of the last dose of DOAC) (b) Tranexamic acid
Rivaroxaban	PT (prolonged), anti-Xa (specific)	None (andexanet withdrawn)	4F-PCC (25–50 U/kg) or aPCC (50–80 U/kg)	Consider: (a) Charcoal (if within 2 h of the last dose of DOAC) (b) Tranexamic acid
Edoxaban	PT, anti-Xa (specific)	None	4F-PCC (25–50 U/kg) or aPCC (50–80 U/kg)	Consider: (a) Charcoal (if within 2 h of the last dose of DOAC) (b) Tranexamic acid

**Legend:** PT: Prothrombin time. INR: International Normalized Ratio. aPTT: activated partial thromboplastin time. FFP: fresh frozen plasma. 4F-PCC: 4 factor prothrombin complex concentrate. aPCC: activated prothrombin complex concentrate.

## Data Availability

No new data were created or analyzed in this study. Data sharing is not applicable to this article.

## Supplementary Materials

The following supporting information can be downloaded at: https://www.mdpi.com/article/10.3390/jcm15072597/s1, File S1: Emergency Anticoagulation Reversal, File S2: Peri-operative management of anticoagulation and antiplatelet therapy, File S3: Appropriate use of tranexamic acid (TXA).
