# Supplementary material for: Anticoagulation Stewardship Program in the DOAC Era
Source: J Clin Med. 2026 Mar 29;15(7):2597. doi: 10.3390/jcm15072597 (PMC13073382; doi:10.3390/jcm15072597)
Supplement: Supplementary file 1 [file jcm-15-02597-s001.zip › Supplement File S1.pdf]

NUH Emergency Anticoagulation Reversal

No	Table Of Content	Page Number
1	**Correction of Supratherapeutic Anticoagulation or Bleeding with Warfarin**<br><br>**Correction of Supratherapeutic Anticoagulation of Bleeding with Warfarin**	2-5
2	**Unfractionated Heparin (UFH) Reversal**	6-8
3	**Low Molecular Weight Heparin (LMWH) Reversal**	9-10
4	**Dabigatran Reversal**	11-13
5	**Factor Xa Inhibitors (Rivaroxaban/Apixaban) Reversal**	14
6	**Antiplatelet Agent Reversal**	15-16
7	**Fibrinolytic Agent Reversal**	17
8	**Annex 1: Management of spontaneous ICH *immediate* post thrombolysis for acute ischemic stroke (for patients requiring craniotomy)**	18-19
9	Octaplex (4-factor PCC)<br><br>**Annex 2A: 4-factor PCC : Contraindications**<br>**Annex 2B: 4-factor PCC: Patient counselling on Risks & Costs**<br>**Annex 2C: Reconstitution and Administration Guide of 4-factor PCC (Octaplex)**<br>**Annex 2D: Patient monitoring guide (following 4-factor PCC administration)**	20-25

Developed by
Version 3: Updated Oct 2025

Purpose:

This guide is intended to provide the practitioner with clear principles and strategies for quality patient care and does not establish a fixed set of rules that preempt physician judgment.

**Major bleed (any of the following)**	
Intracranial haemorrhage on CT/MRI	Non-traumatic intra-articular haematoma
Retroperitoneal haemorrhage on CT/MRI	Any invasive procedure to stop bleeding
Muscle haematoma with compartment syndrome	Any active bleeding from any site plus either SBP<90 mmHg, oliguria or ≥2 g/dl Hb drop
Intra-ocular (non-conjunctival)	Pericardial tamponade
Minor bleed	
Any other bleeding that does not influence the decision to anti-coagulate	

Correction of Supratherapeutic Anticoagulation or Bleeding with Warfarin

Management of warfarin reversal and bleeding events is summarized below:

1. Management of life-threatening bleeds in patients on warfarin
   - 4-factor Prothrombin Complex Concentrate (PCC) is first line unless otherwise contraindicated
   - Each dose of 4-factor PCC will be rounded to the nearest vial size

2. The responsibility of the doctor
   - Ensure patient is on warfarin and has been compliant with it
   - Ensure INR is obtained
   - For a patient known to be compliant with warfarin intake, administration of 4-factor PCC should not be delayed for INR results
   - Repeat INR within 30 mins after administration of 4-factor PCC to monitor for reversal effects
   - High cost form filled up

3. The responsibility of the nurse
   - Administer the product within one hour of preparation [Refer to Annex 2 for 4-factor PCC (Octaplex®) Reconstitution and Administration Guide]

INR	Bleeding	Risk Factors for Bleeding*	Intervention	Monitoring
Supratherapeutic, but <5	No	Regardless	Omit next warfarin dose (s)<br>Consider reducing subsequent dose (s) when restarting	Recheck INR the next day
5-10	No	No	Omit next warfarin dose (s)<br>Consider reducing subsequent dose (s) when restarting	Recheck INR the next day
5-10	No	Yes	Vitamin K 2 mg PO#<br>Omit next warfarin dose (s)<br>Consider reducing subsequent dose (s) when restarting	Recheck INR the next day
> 10	No	Regardless	Vitamin K 2 mg PO#<br>Omit next warfarin dose (s)<br>Consider reducing subsequent dose (s) when restarting	Recheck INR the next day
Minor bleed regardless of INR	Yes		Vitamin K 2mg IV#<br>Omit warfarin	Recheck INR the next day or earlier if clinical deterioration
Major bleed	Yes		Vitamin K 10 mg IV + 4-factor PCC #,ⱡ<br>Omit warfarin<br><br>*Refer to dosing table in the next page for 4-factor PCC dosing*	Recheck INR 30 mins after 4-factor PCC administration, consult Haematology if not corrected<br><br>Due to short half-life of PCC, check INR 6-8 hrs later

***Risk factors for bleeding**:-
Age >65
Prior stroke
Previous severe hemorrhage
Renal dysfunction
Hepatic dysfunction
Anemia
Bleeding disorders (e.g coagulation defect, thrombocytopenia)

#Patients with mechanical prosthetic heart valves are at high risk of valve thrombosis and thromboemboli. For these patients, consult advice must be obtained from Cardiology and Haematology, before warfarin reversal. The risk varies according to valve position and type.

ⱡContraindications to PCC:

- Known allergies to heparin
- Heparin-induced thrombocytopenia

Recommended 4-factor PCC dose banding using both weight and INR (for approx. INR target 1.5)

If the INR is unknown, administrating 1500-2000 IU of PCC can be considered.				
**Weight (kg)**	PCC dose if INR > 6 (40 units/kg)	PCC dose if INR 3-6 (30 units/kg)	PCC dose if INR 2-2.9 (20 units/kg)	PCC dose if INR 1.5-1.9 (10 units/kg)
35-37.9	1500IU	1000IU	500IU	500IU
38-41.9	1500IU	1000IU	1000IU	500IU
42-43.9	1500IU	1500IU	1000IU	500IU
44-56.9	2000IU	1500IU	1000IU	500IU
57-58.9	2500IU	1500IU	1000IU	500IU
59-62.9	2500IU	2000IU	1000IU	1000U
63-68.9	2500IU	2000IU	1500IU	1000U
69-75.9	3000IU*	2000IU	1500IU	1000U
76-87.9	3000IU*	2500IU	1500IU	1000U
88-91.9	3000IU*	2500IU	2000IU	1000U
92-112.9	3000IU*	3000IU*	2000IU	1000U
113-136.9	3000IU*	3000IU*	2500IU	1500U
≥137	3000IU*	3000IU*	3000IU*	1500U

SINGLE DOSES SHOULD NOT EXCEED 3000 UNITS.

[Refer to Annex 2A-2D for more information on 4-factor PCC]

Continued next page→

Reference:
Curtis R et al, Reversal of warfarin anticoagulation for urgent surgical procedures. Can J Anesth 2015

Additional Information about Warfarin Reversal

1. **Administration of vitamin K**
   - Oral vitamin K
     - Use parenteral preparation. Draw required dose with syringe and administer diluted in water/juice.

   - IV vitamin K
     - Slow bolus: Not exceeding 1mg/minute OR
     - Infusion: Dilute in NS or D5W in a minimum of 50mL and infuse over at least 20 minutes

2. Subcutaneous or intramuscular doses are not recommended.

3. Full effect of vitamin K on warfarin reversal occurs approximately 24 hours after administration. Partial effects may be seen in 6-12 hours.

4. Doses of vitamin K greater than 10 mg are excessive and do not reverse anticoagulation more quickly.

5. If there are contraindications to PCC, give fresh frozen plasma (FFP) at least 15ml/kg.

<u>Unfractionated Heparin (UFH) Reversal</u>

Bleeding Severity	Management Recommendations
Mild	Discontinue UFH
Moderate	*Consider any of the following based on bleeding severity:*<br>• Symptomatic treatment<br>• Mechanical compression<br>• Surgical intervention<br>• Fluid replacement and hemodynamic support<br>• Packed red blood cells transfusion
Severe or Life-threatening bleeding or requiring emergency surgery/procedure	• Protamine sulphate (see below for dose calculation and administration)<br>• Other measures as outlined under "Moderate" bleeding severity

Protamine sulfate is used to reverse the anticoagulant effect of heparin.

<u>Dosage and administration:</u>

- 1 mg of protamine neutralizes approximately 100 units of UFH
- <u>Max single protamine dose is 50mg</u>

1. **<u>IV bolus UFH</u>**
    - **Dose calculation:**

Time since IV bolus UFH administered	Dose calculation for protamine sulphate (<u>Max single protamine dose is 50mg</u>):
Within 60 min	Protamine dose (mg) = UFH dose (units)/100
60-120 min	Protamine dose (mg) = {UFH dose (units)/100} x 0.5
>120 min*	Protamine dose (mg) = {UFH dose (units)/100} x 0.25

* Due to short half-life of UFH, it is unlikely that substantial amount of heparin remains after 240 minutes. Protamine is generally not recommended.

- **Administration:** Administer protamine IV with <u>maximum infusion rate of **5 mg/min**</u> to prevent hypotension and bradycardia.

Continued next page→

2. **Continuous IV UFH infusion**

- Only calculate the total amount of UFH administered over the preceding several hours (past 2 hours) prior to reversal.
  - **Dose calculation:**

    E.g. An IV UFH infusion of 1250units/hr requires approximately 25mg of protamine (**max single dose protamine dose is 50mg)**

    Dose calculation for the preceding past 2 hours:
    1250units x 2hours =2500units
    2500/100=25mg protamine

- **Administration:** Administer protamine IV with maximum infusion rate of **5 mg/min** to prevent hypotension and bradycardia.

3. **IV bolus UFH, followed by continuous IV UFH infusion**

- Within 4 hours of IV bolus UFH whilst on continuous IV UFH infusion

Protamine sulfate dose (mg)	=	Dose calculated based on time since IV bolus UFH administered (point no. 1)
		+
		Dose calculated based on continuous IV UFH infusion (point no. 2)

- > 4 hours of IV bolus UFH whilst on continuous IV UFH infusion

Protamine sulfate dose (mg)	=	Dose calculated based on continuous IV UFH infusion (point no. 2)

- **Max single protamine dose is 50mg**

4. **SC UFH** *(Note: There is no consensus on reversal of SC UFH)*

- **Dose calculation**:
  - 1mg of protamine for every 100units of SC UFH given
    - E.g. If 5000units of SC UFH given
      Dose calculation: 5000/100=50mg protamine
- **Administration:**
  - Give 25mg as a slow IV bolus at maximum rate of 5mg/min, with the remaining diluted in NS or D5W to be given as a continuous infusion over 8-16 hours.

Continued next page→

Monitoring:

For IV bolus/continuous IV UFH infusion:
Check aPTT 15 minutes after protamine administration. If aPTT remains prolonged and bleeding continues, refer to Haematology.

For SC UFH:
Check aPTT 15 minutes after protamine administration.
Half-life is longer with subcutaneous administration of UFH; repeat aPTT every 3 hours until bleeding stops. If aPTT remains prolonged and bleeding continues, refer to Haematology.

Note:
Increased risk of hypersensitivity reaction, including anaphylaxis, in patients with a fish allergy or prior exposure to protamine (including protamine-containing insulin preparation, e.g. NPH insulin)
Pre-medicate with corticosteroids and antihistamines if at risk for protamine allergy

- Hydrocortisone 50-100 mg IV x 1 over 15 minutes
- Diphenhydramine 50 mg IV/PO x1

References:

1. Makris M et al, Guideline on the management of bleeding in patients on antithrombotic agents. Brit J Haematol 2012.
2. Frontera JA et al, Guideline for reversal of antithrombotics in intracranial hemorrhage. Neurocrit Care 2016.
3. Garcia DA et al. Parenteral Anticoagulants: Antithrombotic Therapy and Prevention of Thrombosis, 9th ed: American College of Chest Physicians Evidence-Based Clinical Practice Guidelines. Chest 2012.
4. MOH Clinical Practice Guidelines, Dec 2011. Management of Poisoning.

Low Molecular Weight Heparin (LMWH) Reversal

Bleeding Severity	Management Recommendations
Mild	Discontinue LMWH
Moderate	*Consider any of the following based on bleeding severity:*<br>• Symptomatic treatment<br>• Mechanical compression<br>• Surgical intervention<br>• Fluid replacement and hemodynamic support<br>• Packed red blood cells transfusion
Severe or Life-threatening bleeding or requiring emergency surgery/procedure	• Protamine sulphate (see below for dose calculation and administration)<br>• Consider rFVIIa for patients with a contraindication to protamine or LMWH-related bleeding refractory to protamine (to consult Haematologist)<br>• Other measures as outlined under "Moderate" bleeding severity

Protamine sulfate may be used as a **partial reversal agent for LMWH** (neutralizes approximately 60% of LMWH's anti-factor Xa activity).

1. **Dose Calculation**

**Last dose of LMWH given**	**Dose**<br>**(Max single protamine dose is 50mg)**
In the previous 8 hours	1mg protamine for every 1 mg (or every 100 anti-Xa units) of LMWH
In the previous 8-12 hours	0.5 mg protamine for every 1mg (or every 100 anti-Xa units) of LMWH
More than 12 hours	Protamine is not recommended and an alternative agent may be needed to obtain hemostasis. If the patient requires other pharmacologic therapy to manage hemorrhagic complications, a Haematology consult is recommended.

2. **Administration**
   - Administer protamine IV with maximum infusion rate of **5 mg/min** to prevent hypotension and bradycardia.
   - Repeat dose 0.5 mg protamine for every 1 mg (or 100 anti-Xa units) of LMWH if bleeding continues or elevated anti-factor Xa activity level after 2-4 hours (Refer to Haematology)

Continued next page→

Note:

Increased risk of hypersensitivity reaction, including anaphylaxis, in patients with a fish allergy or prior exposure to protamine (including protamine-containing insulin preparation,

e.g. NPH insulin)

Pre-medicate with corticosteroids and antihistamines if at risk for protamine allergy

- Hydrocortisone 50-100 mg IV x 1 over 15 minutes
- Diphenhydramine 50 mg IV/PO x1

References:

1. Makris M et al, Guideline on the management of bleeding in patients on antithrombotic agents. Brit J Haematol 2012.
2. Frontera JA et al, Guideline for reversal of antithrombotics in intracranial hemorrhage. Neurocrit Care 2016.
3. Garcia DA et al. Parenteral Anticoagulants: Antithrombotic Therapy and Prevention of Thrombosis, 9th ed: American College of Chest Physicians Evidence-Based Clinical Practice Guidelines. Chest 2012.
4. MOH Clinical Practice Guidelines, Dec 2011. Management of Poisoning.

Dabigatran Reversal

<table>
<tr><th>Bleeding Severity</th><th>Management Recommendations</th></tr>
<tr><td>Mild</td><td>Delay next dose or discontinue dabigatran.</td></tr>
<tr><td>Moderate</td><td>Consider any of the following based on bleeding severity:
• Symptomatic treatment
• Mechanical compression
• Surgical intervention
• Fluid replacement and hemodynamic support
• Blood product transfusion
• Oral activated charcoal (if previous dose ingested within 2 hours);
Dose: 50 g PO x 1 dose (250mL of 200mg/mL liquid charcoal in sorbitol)</td></tr>
<tr><td>Severe or Life-threatening bleeding or requiring emergency surgery/procedure</td><td>Refer to dabigatran reversal protocol using Idarucizumab (Praxbind) in the next page
Praxbind treatment can be used in conjunction with supportive measures as outlined under “Moderate” bleeding severity
(Consult Haematologist if Idarucizumab is not available)

1. Fatal bleeding, and/or
2. Bleeding in a critical area or organ, such as intracranial, intraspinal, intraocular, retroperitoneal, intra-articular or pericardial, or intramuscular with compartment syndrome, and/or
3. Bleeding causing a fall in hemoglobin level of 2 g/dL or more, or leading to transfusion of two or more units of whole blood or red cells.

<u>Definition of emergency surgery/procedure:</u>
Surgery or other invasive procedures that cannot be delayed for at least 8 hours and for which normal haemostasis would be required.</td></tr>
</table>

NUH clinical pathway and reversal for Dabigatran reversal

Adult patient (18 years of age or older) on dabigatran with severe or life-threatening bleeding or requiring emergency surgery/procedure

↓

In **ED** – ED consultant
In **wards** – Haematologist on-call

↓

1) Screens request and determines if it meets criteria for Idarucizumab usage

2) Send dabigatran level (take 2 citrated tubes), PT/INR, aPTT and TT

→ Does not meet criteria – supportive measures

↓

If meets criteria – approves and recommends Idarucizumab dosing

↓

Primary team doctor informs pharmacist to review order and release Idarucizumab

During office hours (Mon-Sat 830AM-530PM): call Inpatient ACC pharmacist

After office hours: call on-call pharmacist

→ Inpatient ACC pharmacist/on-call pharmacist informs ED pharmacy staff to release Idarucizumab

↓

Primary team doctor informs patient/caregiver on high cost

Then, administers Idarucizumab and fills up high cost form

Idarucizumab dose: ***IV 5g (2x2.5g/50ml)***

Idarucizumab administration: ***Admininster intravenously as two consecutive bolus injections of 2.5g each***

← Emergency surgery / procedure → Repeat PT/INR, aPTT, TT within 15 mins and assess clinical response

↓

Repeat PT/INR, aPTT, TT within 15 mins and assess clinical response

Ongoing major bleeding / dabigatran effects deemed still present

↓

Consult haematology

Updated on Feb 2024

Continued next page→

Important information: -

1. Estimated time to haemostasis normalization based on CrCl:
   Normal renal function: 12 – 24 hrs
   CrCl 50 - 80 mL/min: 24 – 36 hrs
   CrCl 30 - 50 mL/min: 36 – 48 hrs
   CrCl < 30 mL/min: ≥ 48 hrs

Important Special Warnings and Precautions: -

1. Hypersensitivity – If an anaphylactic reaction or other serious allergic reactions occurs, administration of Praxbind should be discontinued immediately and appropriate therapy initiated.

2. Hereditary fructose intolerance – Praxbind contains 4g of sorbitol per dose, as an excipient. In patients with hereditary fructose intolerance, parenteral administration of sorbitol has been associated with reports of hypoglycemia, hypophosphatemia, metabolic acidosis, increase in uric acid, acute liver failure with breakdown of excretory and synthetic function, and death. Weigh the risk against potential benefit of such an emergency treatment. If Praxbind is administered in these patients, intensified medical care during Praxbind exposure and within 24 hours of exposure is required.

3. Thromboembolic events - Reversing dabigatran therapy exposes patients to the thrombotic risk of their underlying disease.

Idarucizumab (Praxbind®) Dosage and Administration

Two 50 mL vials (2x2.5g) constitute one complete dose	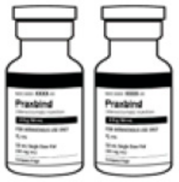
Give the complete 5 g dose in two separate consecutive bolus injections	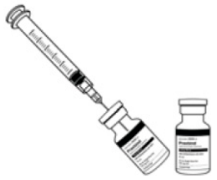

References:
1. Praxbind ® Package Insert, available on HSA website, approved date 30 Aug 2016.
2. Pollack CV Jr et al, Idarucizumab for dabigatran reversal. N Engl J Med 2015.
3. Kaatz S et al, Definition of clinically relevant non-major bleeding in studies of anticoagulants in atrial fibrillation and venous thromboembolic disease in non-surgical patients: communication from the SSC of the ISTH. J Thromb Haemost 2015.

Factor Xa Inhibitors (Rivaroxaban/Apixaban) Reversal

There is no specific reversal agent or pharmacologic antidote, thus management of hemorrhagic complications is primarily supportive. Rivaroxaban and apixaban are highly protein bound and are not dialyzable. If patients require pharmacologic therapy to manage hemorrhagic complications, a Hematology consult is advised. Management of Factor Xa inhibitor-related bleeding events is summarized below:

Bleeding Severity	Management Recommendations
Mild	Delay next dose or discontinue Factor Xa inhibitor.
Moderate	*Consider any of the following based on bleeding severity:*<br>• Symptomatic treatment<br>• Mechanical compression<br>• Surgical intervention<br>• Fluid replacement and hemodynamic support<br>• Blood product transfusion<br>• Oral activated charcoal (if previous dose ingested within 2 hours);<br>**Dose: 50 g PO x 1 dose (250mL of 200mg/mL liquid charcoal in sorbitol)**
Severe or Life-threatening bleeding or requiring emergency surgery/procedure	• Consider any of the strategies outlined above based on bleeding severity.<br>• Consider 4-factor PCC (Octaplex) ɫ.<br>Administer 4-factor PCC 50 units/kg IV x 1. The single dose should not exceed 3000 units. For dose exceeding 3000 units, it can be given as repeated dosing. Each dose of 4-factor PCC to be rounded to the nearest vial.<br>• To investigate potential causes of the bleeding event, obtain the following:- Serum Creatinine, PT, aPTT, FBC, Apixaban/Rivaroxaban anti-Xa level

[Refer to Annex 2A-2D for more information on 4-factor PCC]

ɫContraindications to PCC:

- Known allergies to heparin
- Heparin-induced thrombocytopenia

Important information: -

1. Estimated time to haemostasis normalization based on CrCl:
   Normal renal function: 12 – 24 hrs
   CrCl 50 - 80 mL/min: 24 – 36 hrs
   CrCl 30 - 50 mL/min: 36 – 48 hrs
   CrCl < 30 mL/min: ≥ 48 hrs

References:

1. Eerenberg ES et al, Reversal of Rivaroxaban and Dabigatran by Prothrombin Complex Concentrate. Circulation 2011.
2. Heidbuchel H et al, Updated European Heart Rhythm Association Practical Guide on the use of non-vitamin K antagonist anticoagulants in patients with non-valvular atrial fibrillation. Europace 2015.

Antiplatelet Agent Reversal

Antiplatelet agents have short plasma half-lives, but agents that irreversibly inhibit platelet function may have a prolonged biological effect.

1. Time to normal platelet function after drug discontinuation:
   - NSAIDs, Dipyridamole: 24h
   - Aspirin, clopidogrel, prasugrel, ticlopidine: 5-7 days
   - Ticagrelor: 3-5 days

2. Circulating drug or active metabolites can inhibit transfused platelets.

Bleeding Severity	Management Recommendations
Mild	Consider discontinuing anti-platelet agent
Moderate	**Discontinue anti-platelet agent**<br>*Consider any of the following based on bleeding severity:*<br>• Symptomatic treatment<br>• Mechanical compression<br>• Surgical intervention<br>• Fluid replacement and hemodynamic support
Severe or Life-threatening bleeding or requiring emergency surgery/procedure	Platelet infusion up to a single apheresis unit or equivalent may be considered as additional measure for severe critical bleeds, or prevention of bleeds before emergency surgery

3. Management of antiplatelet agent associated bleeding events:
   - There are no specific reversal agents for antiplatelet agents.
   - Treatment of bleeding involves general hemostatic measures.
   - Discontinuation of antiplatelet agents due to a bleeding event must be weighed against the patient's risk of arterial thrombosis. The risk of thrombosis is particularly high within 1 month of receiving a bare metal coronary stent and within 3 months of receiving a drug eluting coronary stent. Premature cessation of dual anti-platelet therapy in these situations can lead to stent thrombosis which can potentially be fatal. Please consult Cardiologist.
   - Antiplatelet agents should be reinstated as soon as hemostasis is obtained.
   - Platelet infusion may be considered as additional measure for severe critical bleeds, or prevention of bleeds before emergency surgery, but it may confer a risk of arterial thrombosis.

- Platelet infusion should **not** be used for spontaneous intracerebral haemorrhage in people taking antiplatelet therapy (Excluding: spontaneous subarachnoid haemorrhage, arteriovenous malformation haemorrhage, subdural haemorrhage; and at the consultant neurosurgeon’s discretion).
- There is paucity of data that desmopressin is an effective option to reverse antiplatelet agents. It can lead to considerable side effects including arterial vasospasm and hyponatraemia.

References:

1. Makris M et al. Guideline on the management of bleeding in patients on antithrombotic agents. British Journal of Haematology 2012.
2. Ortel TL, Perioperative management of patients on chronic antithrombotic therapy. Blood 2012.
3. Baharoglu, M Irem et al. Platelet transfusion versus standard care after acute stroke due to spontaneous cerebral haemorrhage associated with antiplatelet therapy (PATCH): a randomised, open-label, phase 3 trial. The Lancet , Volume 387 , Issue 10038 , 2605 – 2613.
4. Aldhaeefi M et al. Practical Guide for Anticoagulant and Antiplatelet Reversal in Clinical Practice. Pharmacy 2023, 11, 34. https://doi.org/10.3390/pharmacy11010034.

<u>Fibrinolytic Agent Reversal</u>

Alteplase

- Plasma half-life: 4-8 min
- Duration: Fibrinolytic activity persists for up to 1 hour after the end of infusion

Urokinase

- Half-life elimination: 10-20 min (Delayed in patients with liver disease or impaired renal function)
- Duration: Fibrinolytic activity persists for 12-24 hours after the end of infusion

Note:

- There are no evidence-based guidelines that address management of thrombolysis-associated bleeding.
- Many of the therapies used in practice or that are recommended by guidelines are not clearly specific for reversal of fibrinolytic activity.
- In addition, not all intracranial bleeding after stroke can be ascribed to tPA, and it may be that only a subset of those with sICH will benefit from procoagulant therapy.

Management if bleeding within 48 hours of administration

- The infusion of the fibrinolytic agent should be immediately discontinued
- IV tranexemic acid 1g stat, then 1g 8 hourly
- Consider fresh frozen plasma (FFP) 12-15 ml/kg
- Measure fibrinogen before and after treatment
- If there is depletion of fibrinogen <1g/L, administer cryoprecipitate or fibrinogen concentrate
- Further therapy should be guided by results of coagulation tests

Specific management of spontaneous ICH ***immediate*** post thrombolysis for Acute Ischemic Stroke (please refer to Annex B)

Reference:

1. Makris M et al, Guideline on the management of bleeding in patients on antithrombotic agents. Brit J Haematol 2012.

Annex 1: Management of spontaneous ICH *immediate* post thrombolysis for acute ischemic stroke (for patients requiring craniotomy)

1. Stop infusion of thrombolytic agent

2. Assess patients GCS and manage ABC
   - Avoid hypoxia and hypercarbia
   - Consider Intubation if GCS < 8
   - Control BP (SBP < 160 mmHg)

3. Contact Neurosurgery and ICU
   - Urgent CTB and review by NES

4. Send for lab investigations including ROTEM
   - FBC
   - Coagulation panel including Fibrinogen
   - ROTEM

5. Review by NES for craniotomy:
   - Review CTB for extent of bleed
   - Prognostication based on extent of bleed, co morbidity, etc.

6. If planned for craniotomy, give:
   - IV Tranexamic acid 1 gm
   - IV Cryoppt (10 units) or IV Fibrinogen (dose as below)
   - IV 4FPCC 15units/kg or 10ml/kg FFP
   - IV platelet transfusion (if patient was on Aspirin/ Clopidogrel)
   - IV protamine as per heparin reversal protocol (if patient had received any heparin in recent past)
   - Consider seizure prophylaxis for patients at risk (to be discussed on a case by case basis)

6. Review results:
   ROTEM:
   - Prolonged CT- consider FFP or PCC
   - Extem A10 < 35 mm and Fibtem A10 > 8 mm – consider platelet transfusion
   - Extem A10 < 35 mm and Fibtem A10 < 8 mm- consider iv fibrinogen
   - Thrombolysis seen – consider repeat Tranexamic acid dose.

   PT/ aPTT/ INR/ Fibrinogen:
   - Target INR < 1.3
   - Target fibrinogen > 1.6
   - Target Hb > 8.0
   - Target platelets > 100

8. Continue monitoring Coagulation screen/ ROTEM 4 hrly till target achieved there after 8 hrly till 24 hrs.

9. **General care**:
   - Avoid NG tube for 24 hrs

- Avoid urinary catheter at least 1 hr post rt-PA
- Avoid IM injections for 24 hrs
- Strict glucose control with target <10mmol/lit

<u>Dose of Fibrinogen</u>:

If **plasma fibrinogen level** is known:

$$\text{Dose (gm)} = \frac{[\text{Target level (gm/lit)} - \text{measured level (gm/lit)}] \times \text{BW (kg)}}{20}$$

If **plasma fibrinogen level is not kno**wn:

Dose (mg/kg)= 25- 70 mg/kg of body weight

ROTEM based:

$$\text{Dose (gm)} = \frac{[\text{Target FIBTEM MCF (mm)} - \text{measured FIBTEM MCF (mm)}] \times \text{BW (kg)}}{140}$$

References:

1) Yaghi S, Willey JZ, Cucchiara B, et al. Treatment and outcome of hemorrhagic transformation after intravenous alteplase in acute ischemic stroke: a scientific statement for healthcare professionals from AHA/ASA. Stroke.2017; 48:e1-e19. DOI:10.1161
2) Manuela Carvalho et al. Interventional Algorithms for the control of coagulopathic bleeding in surgical, trauma and postpartum settings: Recommendation from the share network group. Clincial and applied Thrombosis/Hemostasis 2016, Vol.22 (2) 121-137.

Annex 2A: 4-factor PCC : Contraindications

Contraindications
• Hypersensitivity to prothrombin complex concentrate (PCC) or any component of the formulation.<br>• Known allergy to heparin or history of heparin-induced thrombocytopenia (HIT)<br>• History of disseminated intravascular coagulation (DIC)<br>• Individuals who have IgA deficiency with known antibodies against IgA.<br>• Thromboembolic event, myocardial infarction, DIC, cerebral vascular accident, transient ischemic attack, unstable angina pectoris, or severe peripheral vascular disease within the prior 3 months<br>• Labor, obstetric delivery, pregnancy: PCC effect on the fetus is unknown - it is not recommended to use PCC in pregnant patients or during labour unless clearly indicated and benefits outweigh the risk

Annex 2B: 4-factor PCC: Patient counselling on Risks & Costs

**Warnings/Precautions and Monitoring Parameters**
1. Allergic or anaphylactic-type reactions<br>2. Risk of thrombosis (reported to be up to 8% for warfarin reversal; 3.6%-8% for Factor Xa inhibitors reversal) or disseminated intravascular coagulation<br>3. Heparin-induced thrombocytopenia<br><br>Monitor pulse rate before and during the injection. If a marked increase in the pulse rate occurs, reduce the injection speed or interrupt the administration<br><br>Monitor for hypersensitivity or allergic type of reactions (which may include angioedema, injection site reactions, chills, flushing, urticaria, headache, changes in blood pressure, anxiety, nausea, vomiting, sweating, tachycardia, dyspnea, or bronchospasm).<br>If these reactions occur, it is advisable to stop the infusion and contact the doctor immediately.
**Cost**
For >$1000 per course of treatment, fill up High-Cost Drug Treatment Request Form

References:

1) Rodrigues A et al. Urgent Reversal of Direct Oral Anticoagulants in Critical and Life-Threatening Bleeding: A Multidisciplinary Expert Consensus. J. Clin. Med. 2024.
2) Quinlan DJ et al. Four-Factor Prothrombin Complex Concentrate for Urgent Reversal of Vitamin K Antagonistst in Patients With Major Bleeding. Circulation 2013; 128:1179-1181.

Annex 2C: Reconstitution and Administration Guide of 4-factor PCC (Octaplex)

- Each Octaplex vial contains 500IU

Instructions for reconstitution and administration

https://nuh-intranet/Guides-and-Templates/Documents/Pharmacy/Octaplex Nextaro Reconstitution Video.mp4

- **Visually inspect vial for particulate matter and discoloration prior to administration. Do not use solutions that are cloudy or have deposits.**
- **No blood must flow into the syringe due to the risk of formation of fibrin clot.**
- **Octaplex is for single use only.**
- **Record the lot number of the Octaplex vials used in the Nursing Notes.**

Fig. 1

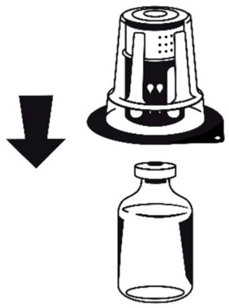

Solvent vial

Fig. 2

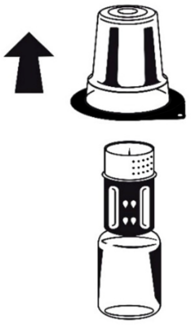

Solvent vial

Fig. 3

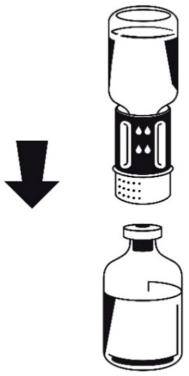

Solvent vial

Powder vial

Fig. 4

Instruction for reconstitution:

1. If necessary, allow the solvent (Water for Injections) and the powder in the closed vials to reach room temperature. This temperature should be maintained during reconstitution.
   If a water bath is used for warming, care must be taken to avoid water coming into contact with the rubber stoppers or the caps of the vials. The temperature of the water bath should not exceed 37°C.

2. Remove the flip off caps from the powder vial and the solvent vial and disinfect the rubber stoppers appropriately.

3. Peel away the lid of the outer package of the Nextaro®. Place the solvent vial on an even surface and hold it firmly. Without removing the outer package, place the blue part of the Nextaro® on top of the solvent vial and press firmly down until it snaps (Fig. 1). Do not twist while attaching! While holding onto the solvent vial, carefully remove the outer package from the Nextaro®, being careful to leave the Nextaro® attached firmly to the solvent vial (Fig. 2).

4. Place the powder vial on an even surface and hold it firmly. Take the solvent vial with the attached Nextaro® and turn it upside down. Place the white part of the Nextaro® connector on top of the powder vial and press firmly down until it snaps (Fig. 3). Do not twist while attaching! The solvent flows automatically into the powder vial.

5. With both vials still attached, gently swirl the powder vial until the product is dissolved. Octaplex dissolves quickly at room temperature to a colourless to slightly blue solution. Unscrew the Nextaro® into two parts (Fig. 4).
   Dispose the empty solvent vial with the blue part of the Nextaro®.

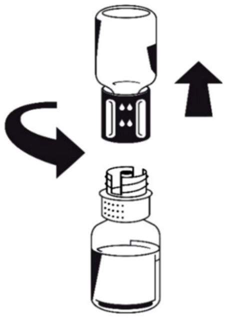	**If the powder fails to dissolve completely or an aggregate is formed, do not use the preparation.**<br><br>6. Attach a 20 mL (500 IU) syringe to the luer lock outlet on the white part of the Nextaro®. Turn the vial upside down and draw the solution into the syringe. Once the solution has been transferred, firmly hold the plunger of the syringe (keeping it facing down) and remove the syringe from the Nextaro®. Dispose the Nextaro® and the empty vial.<br><br>7. Disinfect the intended injection site appropriately.<br><br>8. Inject the solution intravenously at a slow speed: Initially 1 mL/min, not faster than 2 - 3 mL/min (i.e. each vial over minimum of 7 minutes).

Incompatibilities

This product must not be mixed with other medicinal products.

Storage

Store at or below 30°C. Do not freeze.
Store in the original package to protect from light.

After reconstitution the solution must be used immediately. However, if it is not administered immediately, the reconstituted solution can be stored for up to 8 hours at +2°C to +25°C, provided sterility of the stored product is maintained.

Trouble shooting

Reconstitution when Water for Injection (WFI) does not flow

1. If the water for injection (WFI) does not flow when you press down the vial, then remove the WFI vial by unscrewing the blue part of the Nextaro® device as per below:

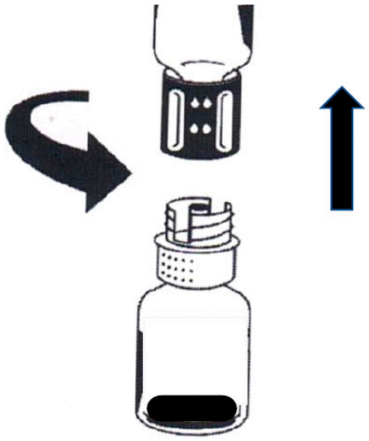

2. Using a new syringe, syringe out 20 mL of the hospital's own water for injection (WFI).

3. Attach the syringe using the luer lock, pump in the 20 mL water for injection into the powder vial.

4. Swirl gently to reconstitute until the powder has dissolved.

5. Attach a 20 mL syringe to the luer lock outlet on the white part of the Nextaro®. Turn the vial upside down and draw the solution into the syringe. Once the solution has been transferred, firmly hold the plunger of the syringe (keeping it facing down) and remove the syringe from the Nextaro®. Dispose the Nextaro® and the empty vial.

For enquiry regarding administration, please contact ward pharmacist during office hour, or on-call pharmacist after office hour.

For emergency issues you may contact Haematologist on-call.

Annex 2D: Patient monitoring guide (following 4-factor PCC administration)

Monitoring Parameters
Monitor pulse rate before and during the injection. If a marked increase in the pulse rate occurs, reduce the injection speed or interrupt the administration.<br><br>Monitor for hypersensitivity or allergic type of reactions (which may include angioedema, injection site reactions, chills, flushing, urticaria, headache, changes in blood pressure, anxiety, nausea, vomiting, sweating, tachycardia, dyspnea, or bronchospasm).<br>If these reactions occur, it is advisable to stop the infusion and contact the doctor immediately.
