# Supplementary material for: Anticoagulation Stewardship Program in the DOAC Era
Source: J Clin Med. 2026 Mar 29;15(7):2597. doi: 10.3390/jcm15072597 (PMC13073382; doi:10.3390/jcm15072597)
Supplement: Supplementary file 1 [file jcm-15-02597-s001.zip › Supplement File S2.pptx]

## Slide 1
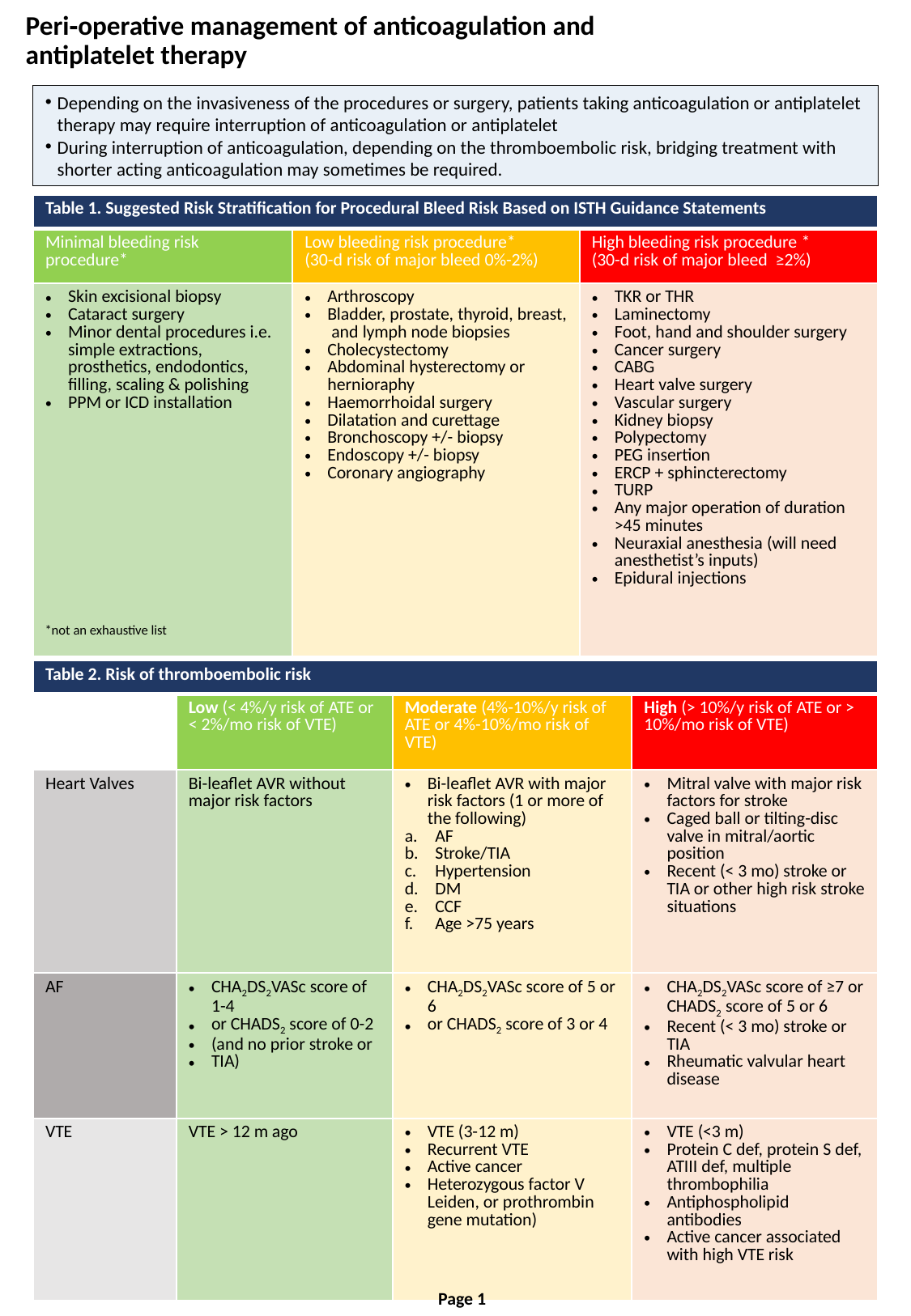

# Peri‐operative management of anticoagulation and antiplatelet therapy
Depending on the invasiveness of the procedures or surgery, patients taking anticoagulation or antiplatelet therapy may require interruption of anticoagulation or antiplatelet
During interruption of anticoagulation, depending on the thromboembolic risk, bridging treatment with shorter acting anticoagulation may sometimes be required.
| Table 1. Suggested Risk Stratification for Procedural Bleed Risk Based on ISTH Guidance Statements | | |
| --- | --- | --- |
| Minimal bleeding risk procedure\* | Low bleeding risk procedure\* (30-d risk of major bleed 0%-2%) | High bleeding risk procedure \* (30-d risk of major bleed ≥2%) |
| Skin excisional biopsy Cataract surgery Minor dental procedures i.e. simple extractions, prosthetics, endodontics, filling, scaling & polishing PPM or ICD installation | Arthroscopy Bladder, prostate, thyroid, breast, and lymph node biopsies Cholecystectomy Abdominal hysterectomy or hernioraphy Haemorrhoidal surgery Dilatation and curettage Bronchoscopy +/- biopsy Endoscopy +/- biopsy Coronary angiography | TKR or THR Laminectomy Foot, hand and shoulder surgery Cancer surgery CABG Heart valve surgery Vascular surgery Kidney biopsy Polypectomy PEG insertion ERCP + sphincterectomy TURP Any major operation of duration >45 minutes Neuraxial anesthesia (will need anesthetist’s inputs) Epidural injections |
*not an exhaustive list
| Table 2. Risk of thromboembolic risk | | | |
| --- | --- | --- | --- |
| | Low (< 4%/y risk of ATE or < 2%/mo risk of VTE) | Moderate (4%-10%/y risk of ATE or 4%-10%/mo risk of VTE) | High (> 10%/y risk of ATE or > 10%/mo risk of VTE) |
| Heart Valves | Bi-leaflet AVR without major risk factors | Bi-leaflet AVR with major risk factors (1 or more of the following) AF Stroke/TIA Hypertension DM CCF Age >75 years | Mitral valve with major risk factors for stroke Caged ball or tilting-disc valve in mitral/aortic position Recent (< 3 mo) stroke or TIA or other high risk stroke situations |
| AF | CHA2DS2VASc score of 1-4 or CHADS2 score of 0-2 (and no prior stroke or TIA) | CHA2DS2VASc score of 5 or 6 or CHADS2 score of 3 or 4 | CHA2DS2VASc score of ≥7 or CHADS2 score of 5 or 6 Recent (< 3 mo) stroke or TIA Rheumatic valvular heart disease |
| VTE | VTE > 12 m ago | VTE (3-12 m) Recurrent VTE Active cancer Heterozygous factor V Leiden, or prothrombin gene mutation) | VTE (<3 m) Protein C def, protein S def, ATIII def, multiple thrombophilia Antiphospholipid antibodies Active cancer associated with high VTE risk |
Page 1

## Slide 2
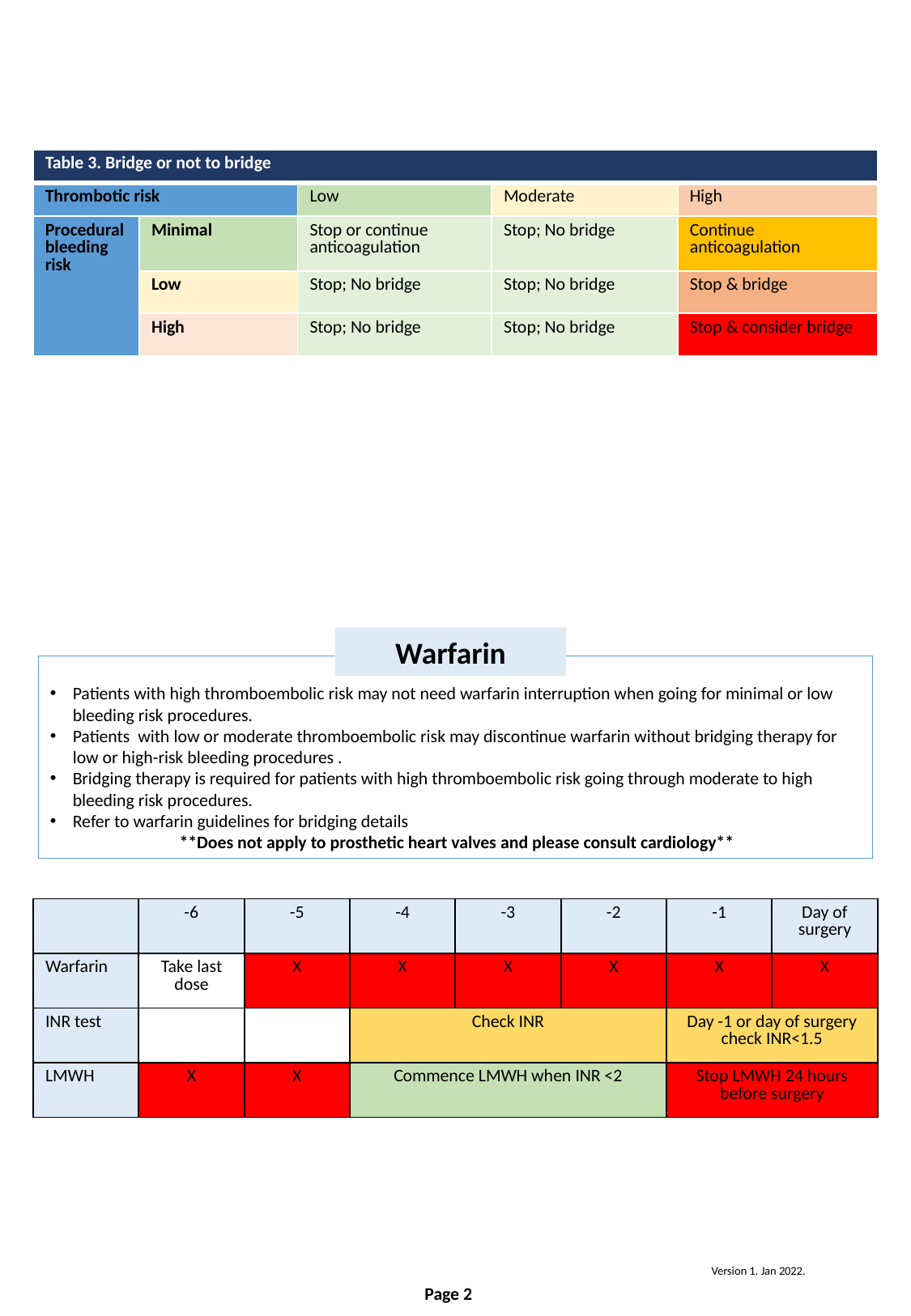

| Table 3. Bridge or not to bridge | | | | |
| --- | --- | --- | --- | --- |
| Thrombotic risk | | Low | Moderate | High |
| Procedural bleeding risk | Minimal | Stop or continue anticoagulation | Stop; No bridge | Continue anticoagulation |
| | Low | Stop; No bridge | Stop; No bridge | Stop & bridge |
| | High | Stop; No bridge | Stop; No bridge | Stop & consider bridge |
Warfarin
Patients with high thromboembolic risk may not need warfarin interruption when going for minimal or low bleeding risk procedures.
Patients with low or moderate thromboembolic risk may discontinue warfarin without bridging therapy for low or high-risk bleeding procedures .
Bridging therapy is required for patients with high thromboembolic risk going through moderate to high bleeding risk procedures.
Refer to warfarin guidelines for bridging details
 **Does not apply to prosthetic heart valves and please consult cardiology**
| | -6 | -5 | -4 | -3 | -2 | -1 | Day of surgery |
| --- | --- | --- | --- | --- | --- | --- | --- |
| Warfarin | Take last dose | X | X | X | X | X | X |
| INR test | | | Check INR | | | Day -1 or day of surgery check INR<1.5 | |
| LMWH | X | X | Commence LMWH when INR <2 | | | Stop LMWH 24 hours before surgery | |
Version 1. Jan 2022.
Page 2

## Slide 3
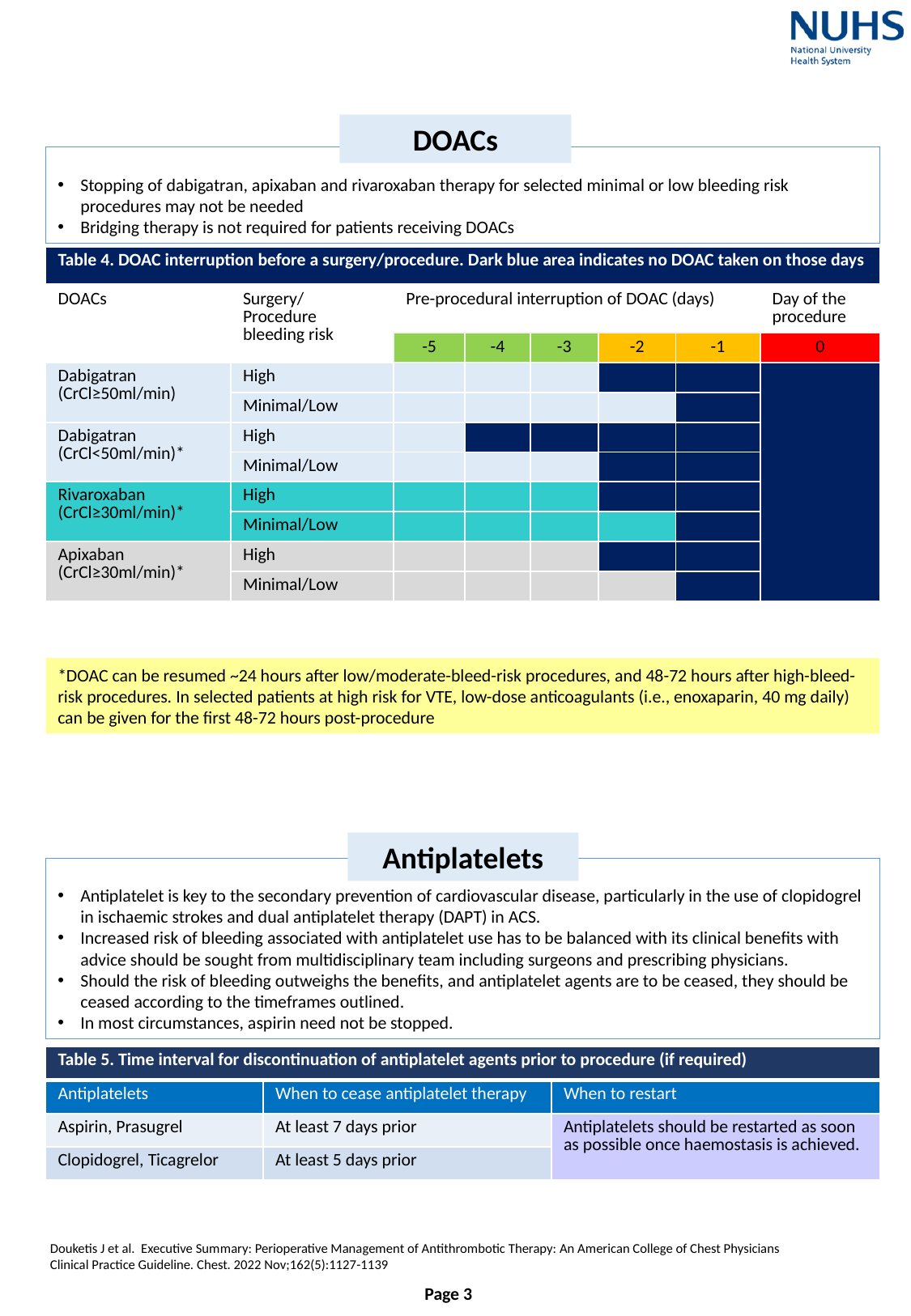

DOACs
Stopping of dabigatran, apixaban and rivaroxaban therapy for selected minimal or low bleeding risk procedures may not be needed
Bridging therapy is not required for patients receiving DOACs
| Table 4. DOAC interruption before a surgery/procedure. Dark blue area indicates no DOAC taken on those days | | | | | | | |
| --- | --- | --- | --- | --- | --- | --- | --- |
| DOACs | Surgery/ Procedure bleeding risk | Pre-procedural interruption of DOAC (days) | | | | | Day of the procedure |
| | | -5 | -4 | -3 | -2 | -1 | 0 |
| Dabigatran (CrCl≥50ml/min) | High | | | | | | |
| | Minimal/Low | | | | | | |
| Dabigatran (CrCl<50ml/min)\* | High | | | | | | |
| | Minimal/Low | | | | | | |
| Rivaroxaban (CrCl≥30ml/min)\* | High | | | | | | |
| | Minimal/Low | | | | | | |
| Apixaban (CrCl≥30ml/min)\* | High | | | | | | |
| | Minimal/Low | | | | | | |
*DOAC can be resumed ~24 hours after low/moderate-bleed-risk procedures, and 48-72 hours after high-bleed-risk procedures. In selected patients at high risk for VTE, low-dose anticoagulants (i.e., enoxaparin, 40 mg daily) can be given for the first 48-72 hours post-procedure
Antiplatelets
Antiplatelet is key to the secondary prevention of cardiovascular disease, particularly in the use of clopidogrel in ischaemic strokes and dual antiplatelet therapy (DAPT) in ACS.
Increased risk of bleeding associated with antiplatelet use has to be balanced with its clinical benefits with advice should be sought from multidisciplinary team including surgeons and prescribing physicians.
Should the risk of bleeding outweighs the benefits, and antiplatelet agents are to be ceased, they should be ceased according to the timeframes outlined.
In most circumstances, aspirin need not be stopped.
| Table 5. Time interval for discontinuation of antiplatelet agents prior to procedure (if required) | | |
| --- | --- | --- |
| Antiplatelets | When to cease antiplatelet therapy | When to restart |
| Aspirin, Prasugrel | At least 7 days prior | Antiplatelets should be restarted as soon as possible once haemostasis is achieved. |
| Clopidogrel, Ticagrelor | At least 5 days prior | |
Douketis J et al. Executive Summary: Perioperative Management of Antithrombotic Therapy: An American College of Chest Physicians Clinical Practice Guideline. Chest. 2022 Nov;162(5):1127-1139
Page 3

## Slide 4
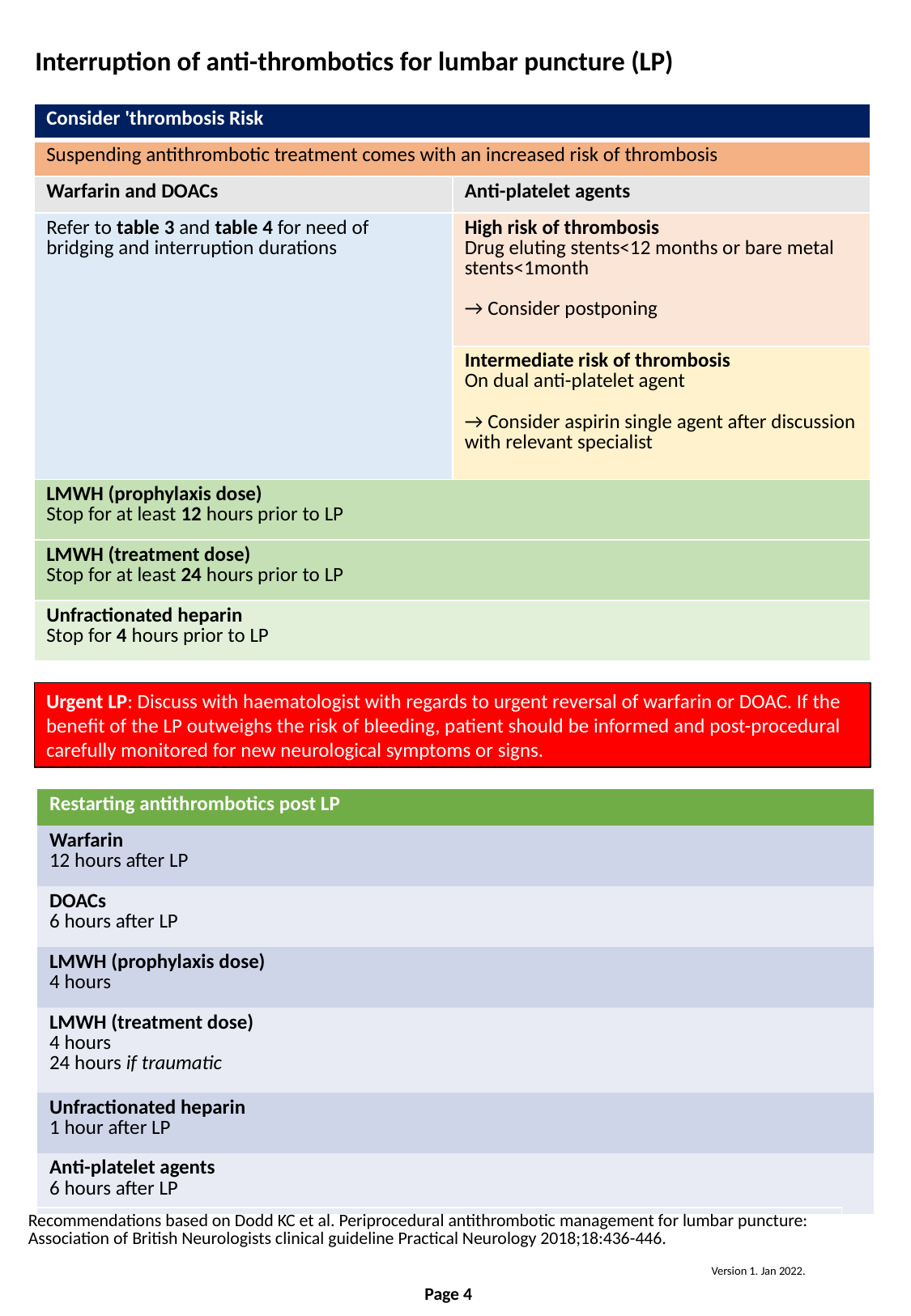

Interruption of anti-thrombotics for lumbar puncture (LP)
| Consider 'thrombosis Risk | |
| --- | --- |
| Suspending antithrombotic treatment comes with an increased risk of thrombosis | |
| Warfarin and DOACs | Anti-platelet agents |
| Refer to table 3 and table 4 for need of bridging and interruption durations | High risk of thrombosis Drug eluting stents<12 months or bare metal stents<1month → Consider postponing |
| | Intermediate risk of thrombosis On dual anti-platelet agent → Consider aspirin single agent after discussion with relevant specialist |
| LMWH (prophylaxis dose) Stop for at least 12 hours prior to LP | |
| LMWH (treatment dose) Stop for at least 24 hours prior to LP | |
| Unfractionated heparin Stop for 4 hours prior to LP | |
Urgent LP: Discuss with haematologist with regards to urgent reversal of warfarin or DOAC. If the benefit of the LP outweighs the risk of bleeding, patient should be informed and post-procedural carefully monitored for new neurological symptoms or signs.
| Restarting antithrombotics post LP |
| --- |
| Warfarin 12 hours after LP |
| DOACs 6 hours after LP |
| LMWH (prophylaxis dose) 4 hours |
| LMWH (treatment dose) 4 hours 24 hours if traumatic |
| Unfractionated heparin 1 hour after LP |
| Anti-platelet agents 6 hours after LP |
| Recommendations based on Dodd KC et al. Periprocedural antithrombotic management for lumbar puncture: Association of British Neurologists clinical guideline Practical Neurology 2018;18:436-446. |
| --- |
Version 1. Jan 2022.
Page 4
