# Supplementary material for: Anticoagulation Stewardship Program in the DOAC Era
Source: J Clin Med. 2026 Mar 29;15(7):2597. doi: 10.3390/jcm15072597 (PMC13073382; doi:10.3390/jcm15072597)
Supplement: Supplementary file 1 [file jcm-15-02597-s001.zip › Supplement File S3.pdf]

GROUP CLINICAL PRACTICE GUIDELINE

APPROPRIATE USE OF TRANEXAMIC ACID (TXA)

Details of Amendments:
New release, not applicable.

1.0 Objectives

1.1 To provide recommendation for the clinical use of tranexamic acid (TXA) in NUHS.

1.2 TXA is a synthetic analog of the amino acid lysine. It is an antifibrinolytic medication that inhibits fibrinolysis by inhibiting the conversion of plasminogen to plasmin, thereby preventing fibrin clot degradation. TXA has been shown to reduce and prevent excessive blood loss in many bleeding scenarios.

1.3 TXA is in the World Health Organization (WHO) Model List of Essential Medicines since 2011 and is recommended by WHO for the treatment of acute bleeding in trauma, cardiopulmonary bypass and postpartum haemorrhage.

2.0 Scope

2.1 The use of TXA for the clinical presentations are recommended based on the clinical trials and literature cited within this CPG.

2.2 TXA use is shown to improve haemostasis in many types of surgeries and lower rates of blood transfusion. Additionally, the use of TXA does not increase the risk of vascular adverse events and death.

2.3 This CPG applies to medical staff, nurses and pharmacists who are involved in care of patients who may require TXA.

3.0 Standards/ Regulations

3.1 NIL

4.0 References

4.1 CRASH-2 trial collaborators, Shakur H, Roberts I, Bautista R, Caballero J, Coats T, et al. Effects of tranexamic acid on death, vascular occlusive events, and blood transfusion in trauma patients with significant haemorrhage (CRASH-2): a randomised, placebo-controlled trial. Lancet. 2010;376(9734):23–32. Epub 18 Jun 2010

4.2 Roberts I, et al. “Effects of tranexamic acid on death, disability, vascular occlusive events and other morbidities in patients with acute traumatic brain injury (CRASH-3): a randomized, placebo-controlled trial”. The Lancet. 2019. 394(10210):1713-1723

4.3 Shakur, Haleema et al. Effect of early tranexamic acid administration on mortality, hysterectomy, and other morbidities in women with post-partum haemorrhage (WOMAN): an international, randomised, double-blind, placebo-controlled trial. The Lancet, Volume 389, Issue 10084, 2105 – 2116

4.4 Guo, J., Gao, X., Ma, Y. et al. Different dose regimes and administration methods of tranexamic acid in cardiac surgery: a meta-analysis of randomized trials. BMC Anesthesiol 19, 129 (2019). https://doi.org/10.1186/s12871-019-0772-0

4.5 Shi J, et al. Effect of High- vs Low-Dose Tranexamic Acid Infusion on Need for Red Blood Cell Transfusion and Adverse Events in Patients Undergoing Cardiac Surgery:

The OPTIMAL Randomized Clinical Trial. JAMA. 2022 Jul 26;328(4):336-347.

4.6 Sprigg N et al. TICH-2 Investigators. Tranexamic acid for hyperacute primary IntraCerebral Haemorrhage (TICH-2): an international randomised, placebo-controlled, phase 3 superiority trial. Lancet. 2018 May 26;391(10135):2107-2115.

4.7 Roberts, Ian et al. Effects of a high-dose 24-h infusion of tranexamic acid on death and thromboembolic events in patients with acute gastrointestinal bleeding (HALT-IT): an international randomised, double-blind, placebo-controlled trial. The Lancet, Volume 395, Issue 10241, 1927 – 1936

4.8 Devereaux PJ, et al. POISE-3 Investigators. Tranexamic Acid in Patients Undergoing Noncardiac Surgery. N Engl J Med. 2022 May 26;386(21):1986-1997.

4.9 UK NICE National Institute for Health and Care Excellence. Blood Transfusion Quality Standard. 15 Dec 2016. https://www.nice.org.uk/guidance/qs138

4.10 Grocott M P W et al. The UK Royal Colleges Tranexamic Acid in Surgery Implementation Group. Tranexamic acid for safer surgery: the time is now. British Journal of Surgery. 2022 Dec;109(12): 1182–1183

4.11 Jerath A, et al. Tranexamic Acid Dosing for Cardiac Surgical Patients With Chronic Renal Dysfunction: A New Dosing Regimen. Anesth Analg. 2018 Dec;127(6):1323-1332.

4.12 Yang, QJ et al. Pharmacokinetic modeling of tranexamic acid for patients undergoing cardiac surgery with normal renal function and model simulations for patients with renal impairment. Biopharm. Drug Dispos., 2015;35, 294–307.

4.13 Tranexamic acid. In: MerativeTM Micromedex® DRUGDEX® (electronic version). Merative, Ann Arbor, Michigan, USA. Available at: https://www.micromedexsolutions.com/. Accessed 8 July 2024.

5.0 Guiding Principles

5.1 NIL

6.0 Definitions

6.1 IV – Intravenous

6.2 OR – Lack of/ without

6.3 PO – Oral Administration

6.4 QDS – Four times a day

6.5 TDS – Three times a day

6.6 TXA – Tranexamic Acid

7.0 Details of Guideline

7.1 Clinical Indications and Recommended TXA Regimen

7.1.1 The use of TXA is recommended in the following clinical presentations:

(a) Trauma-associated hemorrhage

(b) Traumatic brain injury with GCS > 9

(c) Postpartum hemorrhage

(d) Reducing transfusion in cardiac surgery

(e) Reducing blood loss for surgeries with anticipated blood loss of >500ml

(f) Reducing transfusion during orthopaedic and spine surgery

(g) Topical surgical field blood loss reduction

(h) Intracerebral Hemorrhage (ICH)

(i) Haemoptysis

(j) Heavy Menstrual Bleeding

(k) Von Willebrand Disease (VWD) related bleeding

(l) Epistaxis

(m) Hereditary Hemorrhagic Telangiectasia related bleeding

7.1.2 The table below summarises the recommended TXA regimes based on the clinical indication.

S/N	Clinical Indication	TXA Regimen
1	Trauma-associated hemorrhage	1g IV over 10 min then 1g over the next 8 hours as a continuous infusion
2	Traumatic brain injury with GCS > 9	1g IV over 10 min then 1g over the next 8 hours as a continuous infusion
3	Postpartum hemorrhage	1g IV over 10 min. If bleeding continues after 30 min or restarts within 24h a 2nd dose of 1g IV can be given

4	Reducing transfusion in cardiac surgery	Low dose regimen (preferred): 10mg/kg IV over 30 min, followed by 1-2mg/kg/h till end of surgery, *or*<br>1g IV at start of surgery and 1g IV at end of surgery<br><br>High dose regimen: 30mg/kg IV over 30min, followed by 10-16mg/kg/h till end of surgery should only be considered for patients with high risk of bleeding and age <70, normal renal function and non-open chamber surgery.
5	Reducing blood loss for surgeries with anticipated blood loss of >500ml	1g IV at start of surgery and 1g IV at end of surgery
6	Reducing transfusion during orthopaedic and spine surgery	1g IV at start of surgery and 1g IV at end of surgery<br>*or*<br>Loading dose IV 10mg/kg, maintenance 1mg/kg/hr infusion (Specific guidance available – Reference 4.10, 4.11)
7	Topical surgical field blood loss reduction in orthopaedic surgery	Variable doses: most commonly 1g TXA in 50 ml administered intra-articularly
8	Intracerebral Hemorrhage (ICH)	1g IV over 10 min then 1g over the next 8 hours as a continuous infusion
9	Hemoptysis	500-1000mg nebulised in 5-10ml 0.9% normal saline PRN, up to 3 times daily for up to 5 days
10	Heavy Menstrual Bleeding	500-1000mg PO 3 times daily for up to 5 days during each monthly menstruation
11	Von Willebrand Disease (VWD) related bleeding	Oral: TXA 20mg/kg PO TDS<br>Mouthwash for oral bleeding or tooth extraction: TXA 1–1.5g in 10ml QDS prn – Swish and spit
12	Epistaxis	Topical: Cotton gauze soaked with injectable form of TXA (500mg in 5ml)

13	Hereditary Hemorrhagic Telangiectasia related bleeding	1g PO TDS
14	Paediatric trauma and surgical bleeding (age <12 years)	15mg/kg IV (max 1g) over 10min, followed by 2mg/kg/h till end of surgery

7.1.3 This CPG is unable to provide recommendations for TXA use in the following presentations:

(a) Prophylaxis in Acute Myeloid Leukemia related bleeding

(b) Prophylaxis in Acute Promyelocytic Leukemia related bleeding

(c) Melasma

(d) Hereditary angioedema

7.1.4 The use of TXA is suggested against if patient presents with the following:

(a) Empiric treatment of GI bleeding

(b) Use in subarachnoid haemorrhage

(c) Use in chronic thrombocytopenia without bleeding

7.1.5 Surgeries for which intraoperative TXA is recommended for reduction of blood loss:

(a) Orthopaedic – total hip replacement, total knee replacement, hip arthroplasty, hip fracture surgery, major spine surgery

(b) General surgery – colorectal resection, liver resection

(c) Urologic – nephrectomy, cystectomy

(d) Gynaecologic – simple or complex hysterectomy

(e) Cardiac – coronary artery bypass graft, valve replacement, aneurysm repair

(f) Other surgeries with anticipated blood loss of >500ml

7.2 Clinical Complications

7.2.1 Contraindications

(a) The use of TXA is not recommended if the patient has a known anaphylactic reaction/ hypersensitivity to TXA

(b) Disseminated intravascular coagulation (unless hyperfibrinolysis is present)

(c) Haematuria in renal parenchyma disease

7.2.2 Precautions

(a) Recent history of arterial or venous thrombosis (for example, stroke, myocardial infarction, venous thromboembolism) in the last 3 months

(b) Hypercoagulable states

(c) History of seizure disorder

(d) Concomitant administration of activated clotting factor concentrates (FEIBA/ Recombinant activated FVII)

NB: Precautions indicate that TXA should be avoided in elective situations. However, in urgent situations where the benefits of TXA outweigh the risks, such as traumatic haemorrhage or active haemorrhage when there is hyperfibrinolysis, TXA can be used in 1-2 doses.

7.2.3 Side Effects of TXA include:

(a) Headache (50% incidence)

(b) Nasal Symptoms (25% incidence)

(c) Nausea, vomiting, diarrhea, abdominal pain (20% incidence)

(d) Musculoskeletal pain, arthralgia, back pain (10% incidence)

(e) Fatigue (5% incidence)

(f) Impaired, blur and color vision impairment (<1% incidence)

(g) Seizure (<1% incidence) – associated with higher doses. Avoid bolus dose >30mg/kg or total daily dose >50mg/kg

(h) Thrombosis (no convincing evidence at recommended doses)

7.2.4 As urinary excretion is the main route of elimination for TXA, dose adjustment for renal impairment is required.

(a) The table below provides guidelines to adjusting TXA dose (mg/kg) for regular TDS dosing based on serum creatinine (μmol/L).

Serum creatinine	Adjusted TXA Dose for Regular TDS Dosing
120-250 µmol/L	15 mg/kg PO or<br>10 mg/kg IV BID
250-500 µmol/L	15 mg/kg PO or<br>10 mg/kg IV daily
>500 µmol/L	15 mg/kg PO or<br>10 mg/kg q48h
Dialysis	15 mg/kg PO or<br>10 mg/kg q48h

(b) The table below provides guidelines for TXA dose reduction for indications requiring only 1-2 doses or infusion, based on creatinine clearance (mL/min).

Creatinine Clearance	Dose Reduction for indications requiring only 1-2 doses*	Cardiac surgery high dose regimen infusion (Ref. Micromedex)
60 – <90 ml/min	25% dose reduction	10mg/kg/h
30 – <60 ml/min	50% dose reduction	5mg/kg/h
<30 ml/min	Avoid or 75% dose reduction	3mg/kg/h
Dialysis	Avoid or 75% dose reduction	3mg/kg/h

* No dose reduction recommended for trauma or post-partum haemorrhage.

8.0 Compliance Monitoring

8.1 Use of TXA for reducing blood loss in surgery may be monitored from time to time, to encourage a high level of adoption for eligible patients.

9.0 Contact Information

9.1 Please contact your institutional Medical Affairs/ Quality if you have any queries on this clinical practice guideline.

10.0 Annex

10.1 NIL

11.0 Associated Documents

11.1 NIL

12.0 Authors
